# A NIR‐II Photoactivatable “ROS Bomb” with High‐Density Cu_2_O‐Supported MoS_2_ Nanoflowers for Anticancer Therapy

**DOI:** 10.1002/advs.202302208

**Published:** 2023-06-20

**Authors:** Jia Huang, Guiming Deng, Shuya Wang, Tianjiao Zhao, Qiaohui Chen, Yuqi Yang, Yongqi Yang, Jinping Zhang, Yayun Nan, Zhaoqian Liu, Ke Cao, Qiong Huang, Kelong Ai

**Affiliations:** ^1^ Department of Pharmacy Xiangya Hospital Central South University Changsha 410008 China; ^2^ Department of Pharmacology, Xiangya School of Pharmaceutical Sciences Central South University Changsha 410078 China; ^3^ Hunan Provincial Key Laboratory of Cardiovascular Research Xiangya School of Pharmaceutical Sciences Central South University Changsha 410078 China; ^4^ Department of infection and liver disease The First Hospital of Hunan University of Chinese Medicine Changsha 410007 China; ^5^ National Clinical Research Center for Geriatric Disorders Xiangya Hospital Central South University Changsha 410008 China; ^6^ Geriatric Medical Center People's Hospital of Ningxia Hui Autonomous Region Yinchuan Ningxia 750002 China; ^7^ Department of Oncology The Third Xiangya Hospital of Central South University Changsha 410013 China

**Keywords:** chemodynamic therapy, cuprous oxide, molybdenum disulfide, photothermal therapy, synergistic therapy

## Abstract

The fast conversion of hydrogen peroxide (H_2_O_2_) into reactive oxygen species (ROS) at tumor sites is a promising anticancer strategy by manipulating nanomedicines with near‐infrared light in the second region (NIR‐II). However, this strategy is greatly compromised by the powerful antioxidant capacity of tumors and the limited ROS generation rate of nanomedicines. This dilemma mainly stems from the lack of an effective synthesis method to support high‐density copper‐based nanocatalysts on the surface of photothermal nanomaterials. Herein, a multifunctional nanoplatform (MCPQZ) with high–density cuprous (Cu_2_O) supported molybdenum disulfide (MoS_2_) nanoflowers (MC NFs) is developed for the efficient killing of tumors via a potent ROS storm by an innovative method. Under NIR‐II light irradiation, the ROS intensity and maximum reaction velocity (*V*_max_) produced by MC NFs are 21.6 and 33.8 times that of the non–irradiation group in vitro, which is much higher than most current nanomedicines. Moreover, the strong ROS storm in cancer cells is efficiently formed by MCPQZ (increased by 27.8 times compared to the control), thanks to the fact that MCPQZ effectively pre–weakens the multiple antioxidant systems of cancer cells. This work provides a novel insight to solve the bottleneck of ROS‐based cancer therapy.

## Graphical abstract

In response to the limitations of traditional cancer synergistic photothermal–chemodynamic therapy (PTT‐CDT), MoS_2_–Cu_2_O–PEG@QE/Znpp IX (MCPQZ) can pre‐weaken tumor multiple resistance and induce severe cell apoptosis by ·OH‐induced lysosomal membrane penetration under near‐infrared light in the second region laser irradiation, thereby achieving high‐efficient synergistic therapeutic effects.

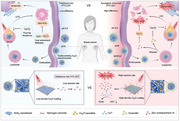

## 1 Introduction

Conversion of oxygen (O_2_) or hydrogen peroxide (H_2_O_2_) into strongly oxidized reactive oxygen species (ROS) is a promising anti‐tumor strategy and serves as the basis for chemodynamic therapy (CDT),^[^ ^1^ ^]^ sonodynamic therapy,^[^ ^2^ ^]^ photodynamic therapy,^[^ ^3^ ^]^ and radiodynamic therapy.^[^ ^4^ ^]^ However, the therapeutic efficacy of these treatments is largely limited by three aspects. First, the strong antioxidant systems of tumors,^[^ ^5^ ^]^ including elevated concentrations of glutathione (GSH) and highly active heme oxygenase‐1 (HO‐1), are highly effective in scavenging ROS.^[^ ^6^ ^]^ Second, the supply of H_2_O_2_ is insufficient for therapy despite the concentration of H_2_O_2_ at tumor sites (50–100 µm) being markedly higher than that in normal cells.^[^ ^7^ ^]^ Third, the ROS generation rate of most nanomedicines is seriously limited. For example, the highest reaction rate of typical Fe‐based nanocatalysts for conversion of H_2_O_2_ to hydroxyl radical (·OH) is only 63 m^−1^ s^−1^ in CDT.^[^ ^8^ ^]^ Although copper‐based nanocatalysts can increase the conversion efficiency to some extent (the highest reaction rate of up to 1 × 10^4^ m^−1^ s^−1^),^[^ ^9^ ^]^ their activity is still far inadequate to generate a ROS storm for cancer therapy. As a result, the antioxidant system of cancer cells can easily remove ROS to lead to poor treatment effects if H_2_O_2_ is not supplied continuously or the reaction rate is not adequately rapid.

Currently, many emerging multifunctional nanoplatforms have been developed to solve these issues. In general, these nanoplatforms are classified into two categories: (1) increasing ROS production through H_2_O_2_ supply or enhancing catalytic activity, such as nanocomposites of gold nanorods and perfluorocarbon,^[^ ^10^ ^]^ palladium nanosheets,^[^ ^11^ ^]^ and ZIF‐8 nanoplatforms loaded with glucose oxidase and horseradish peroxidase (POD);^[^ ^12^ ^]^ (2) disrupting the antioxidant defense system of cancer cells, such as scavenging GSH with gold–manganese oxide nanocomposites.^[^ ^13^ ^]^ Correspondingly, to fulfill these multifunctional characteristics, increasingly complex and sophisticated nanocomposites not only lead to considerable challenges in the preparation process but also suffer from low reproducibility and reliability, severe side‐effects, and high cost.^[^ ^14^ ^]^ In addition, the majority of multifunctional nanoplatforms can only overcome one or two restrictions and therapeutic efficiency thus remains far from satisfactory.^[^ ^15^ ^]^ From the perspective of practical applications, nanoplatforms composed of biodegradable elements in vivo have better prospects.^[^ ^16^ ^]^ Ideally, ROS‐based treatment strategies should initially remove the antioxidant defense system of tumors and convert limited H_2_O_2_ into a ROS storm as rapidly as possible to destroy key organelles and induce cancer cell apoptosis to achieve successful therapeutic effects against solid tumors. However, multifunctional nanoplatforms are difficult to achieve for this ambition since multiple factors need to be considered and various factors are hard to synergistically combine to enhance the anticancer effect. For instance, a promising strategy is to promote the ROS generation rate by loading cuprous oxide (Cu_2_O) on the surface of photothermal nanomaterials through near‐infrared (NIR) light‐induced thermal effects.^[^ ^17^ ^]^ However, the low Cu_2_O loading density leads to a limited increase in the ROS generation rate, which greatly limits the intensity of the ROS storm and results in a largely compromised anticancer effect.

**Scheme 1:**
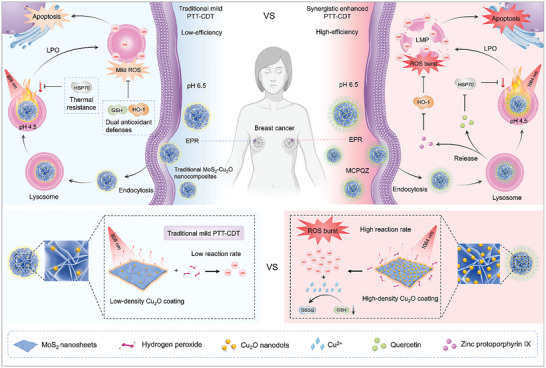
Schematic illustration for the anticancer‐mechanisms of MCPQZ‐mediated cancer synergistic photothermal‐chemodynamic therapy (PTT‐CDT). After internalized into cancer cells and captured by lysosomes, MCPQZ can inhibit the expression of HSP70 and the activity of HO‐1 to break tumor thermal‐resistance and antioxidant defense by the released QE and Znpp IX, respectively. Importantly, compared to MoS_2_/Cu_2_O nanocomposites with low‐density Cu_2_O coating prepared by traditional synthesis methods, our innovative synthesis strategy can in situ grow a high‐density Cu_2_O coating on the surface of MoS_2_ nanoflowers, which is undoubtedly favorable for improving catalytic efficiency of nanocatalysts. Therefore, under 1064 nm laser irradiation, the as‐prepared MCPQZ can act as a photoactivatable nanocatalyst to efficiently transfer hyperthermia generated from photothermal effect to the high‐density Cu_2_O coating in situ, greatly promoting the Cu^+^‐catalyzed Fenton‐like reaction rate and accompany with Cu^2+^‐mediated GSH depletion to elicit ROS burst, thus achieving high‐efficiency PTT‐CDT synergistic effect. Based on this, MCPQZ can cause severe LMP by ·OH‐triggered lysosomal lipid peroxidation (LPO), inducing cancer cell apoptosis.

To address these challenges, herein, we developed a multifunctional nanoplatform (MoS_2_–Cu_2_O–PEG@QE/Znpp IX [MCPQZ]) for efficient killing of tumors via a potent ROS storm based on specially prepared high‐density Cu_2_O‐supported molybdenum disulfide (MoS_2_) nanoflowers (MC NFs) with an innovative method. MC NFs were further adopted as a substrate for co‐loading of quercetin (QE, a heat shock protein 70 [HSP70] inhibitor) and zinc‐protoporphyrin IX (Znpp IX, an HO‐1 inhibitor) to obtain the MCPQZ nanoplatforms. Specifically, QE and Znpp IX are first released from MCPQZ to greatly pre‐weaken the multiple defense systems of cancer cells by efficiently inhibiting HSP70 expression and HO‐1 activity at tumor sites, respectively. Notably, MC NFs, as a high‐efficient second near‐infrared (NIR‐II) photothermal agent (the photothermal conversion [PTC] efficiency is 45.44%), can effectively transfer heat to the superficial high‐density Cu_2_O in situ, which greatly increases the reaction rate of the Cu^+^‐catalyzed Fenton‐like reaction and generates potent ROS burst. Meanwhile, oxidized Cu^2+^ further depletes the high levels of GSH to reduce ROS scavenging. As follow, a strong ROS storm is formed and induces cancer cell apoptosis via ROS‐triggered lysosomal membrane penetration (LMP) (**Scheme** **1**). In vitro and in vivo experiments fully demonstrated that the dual synergistic strategy (destroy the antioxidant system of cancer cells and generate ROS storm) based on MCPQZ can effectively treat cancer. This work provides a novel insight to solve the bottleneck of ROS‐based therapy for cancer.

## 2 Results and Discussion

### 2.1 Synthesis and Characterization of MC NFs

**Figure 1:**
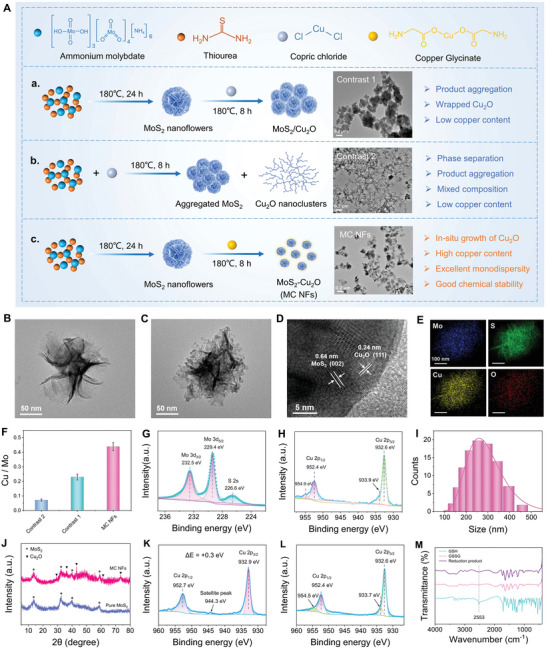
A) Schematic illustration for the synthesis of (a) Contrast 1, (b) Contrast 2, and (c) MC NFs and their corresponding TEM images. B) TEM image of pure MoS_2_. C) TEM image, D) HRTEM image, and E) element mapping of MC NFs. F) The content ratio of Cu and Mo elements (Cu/Mo) of Contrast 1, Contrast 2, and MC NFs estimated by ICP‐OES. Data are defined as mean ± S.D. (*n* = 3). High‐resolution G) Mo 3d and H) Cu 2p XPS spectra of MC NFs. I) Size distribution of MC NFs. J) XRD patterns of pure MoS_2_ and MC NFs. High‐resolution Cu 2p XPS spectra of K) the oxidation product of MC NFs reacted with H_2_O_2_ and L) the reduction product reduced by GSH after reacting with H_2_O_2_. M) FT‐IR spectra of GSH, commercial GSSG, and the reduction product.

Generally, Cu–MoS_2_ nanocomposites are prepared by growing Cu complexes on MoS_2_ with copper salts as precursors. However, copper ions lead to the severe aggregation of negatively charged MoS_2_, making it difficult to form monodisperse Cu–MoS_2_ nanocomposites (Contrast 1, **Figure** **1**A[a] and Figure S1A, Supporting Information). Another preparation method of Cu–MoS_2_ nanocomposites is to add copper salts when preparing MoS_2_ nanoparticles. During this process, Cu ions rapidly react with S ions from the sulfur precursor to self‐nucleate, leading to phase separation of MoS_2_ and Cu composites (Contrast 2, Figure 1A[b] and Figure S2, Supporting Information). In addition, neither of these two methods can achieve high loading of copper complexes on the surface of MoS_2_ nanoparticles. To this end, we prepared high‐density Cu composites‐supported MoS_2_ nanocomposites via two steps (Figure 1A[c]). First, MC NFs were synthesized via a modified hydrothermal method according to our previously published protocol.^[^ ^18^ ^]^ Next, high‐density Cu complexes were grown in situ on the surface of MC NFs with copper glycine as the copper precursor. In this case, the electrically neutral copper glycine effectively prevented precipitation of MoS_2_ nanoparticles and subsequent product agglomeration owing to its uncharged nature. In addition, compared with Cu salts, copper glycinate is more stable during pyrolysis, which avoids the problem of phase separation of MoS_2_ and Cu composites caused by the spontaneous nucleation of copper complexes, and finally forms dense Cu complexes on MC NFs. Transmission electron microscopy (TEM) showed that the as‐prepared MoS_2_ displayed a 3D flower‐like structure consisting of numerous 2D crumpled paper‐like nanosheets (Figure 1B). As shown in Figure 1C, a high‐density of Cu_2_O nanodots grew in situ on the surface of MC NFs. High‐resolution TEM images disclosed lattice spacing of 0.64 and 0.24 nm, which coincident with the (002) and (111) plane of 2H–MoS_2_ and cubic Cu_2_O, respectively (Figure 1D). Elemental mapping revealed a homogeneous distribution of Mo, S, Cu, and O elements on MC NFs (Figure 1E). More importantly, the copper content of MC NFs was significantly higher than that of the other two nanocomposites (Contrast 1 and 2). The content ratio of Cu and Mo elements (Cu/Mo) of MC NFs was 6.16 times that of Contrast 2 and 1.91 times that of Contrast 1 measured by inductively coupled plasma optical emission spectrometry (ICP‐OES) (Figure 1F). In addition, the Cu/Mo values of MC NFs prepared under different feed ratios (the molar mass ratio of Mo and Cu [*n* [Mo]:*n* [Cu]] = 10:1, 10:2, 10:3, 10:4, and 10:5) were also measured by ICP‐OES (Figure S3, Supporting Information). Expectedly, the Cu/Mo values of MC NFs increase as the feed ratio increases. X‐ray electron spectroscopy (XPS) results showed that MC NFs mainly comprise Mo, Cu, S, and O elements (Figure S4A, Supporting Information). The two peaks at 229.4 and 232.5 eV were assigned to Mo 3d_5/2_ and Mo 3d_3/2_ of Mo^4+^ while the two peaks at 162.3 and 163.5 eV were consistent with S 2p_3/2_ and S 2p_1/2_ of S^2−^ (Figure 1G and Figure S4B, Supporting Information). The two characteristic peaks at 932.6 and 952.4 eV were assigned to Cu 2p_3/2_ and Cu 2p_1/2_ of Cu^+^ and the two weak signals at 933.9 and 954.9 eV were assigned to a small proportion of oxidized Cu^2+^ (Figure 1H). Notably, no characteristic satellite peak of Cu (II) was observed between Cu 2p_3/2_ and Cu 2p_1/2_, further confirming that the valence state of Cu in MC NFs is mainly +1.^[^ ^19^ ^]^ The XPS peaks of O1s at 530.8 eV corresponded to Cu—O in Cu_2_O while the weak peak at 533 eV belonged to O—H of surface‐adsorbed H_2_O (Figure S4C, Supporting Information). High‐resolution Cu 2p XPS spectra of Contrast 1 and Contrast 2 exhibited similar patterns as MC NFs, indicating that the valence state of Cu is predominantly +1 (Figure S5, Supporting Information). The average hydrodynamic diameter of MC NFs was also much smaller than that of Contrast 1 (265.8 vs 698.8 nm) by dynamic light scattering (DLS) measure (Figure 1I and Figure S1B, Supporting Information), which benefits from the electrically neutral nature of copper glycinate. In addition, the zeta potential of MC NFs was −27.37 mV (Figure S6, Supporting Information). The negative surface charge enables MC NFs to keep stable in the blood circulation system and reach the tumor site through the enhanced permeability and retention effect. As shown in Figure 1J, X‐ray diffraction (XRD) results indicated that both pure MoS_2_ and MC NFs showed diffraction peaks at 2*θ* of 14°, 33°, 40°, and 59° corresponding to the (002), (100), (103), and (110) plane of 2H–MoS_2_ (JCPDS no. 75‐1539),^[^ ^20^ ^]^ respectively. Notably, diffraction peaks at 29.5°, 36.5°, 42°, 61°, and 73.5° in the XRD pattern of MC NFs were assigned to the (110), (111), (200), (220), and (311) plane of cubic Cu_2_O (JCPDS no. 77‐0199),^[^ ^21^ ^]^ respectively. Overall, MC NFs with excellent monodispersity, good chemical stability, and high copper content were successfully formulated.

To confirm our hypothesis that cascade reactions occur among Cu^+^, H_2_O_2_, Cu^2+^, and GSH, equivalent molar masses of MC NFs and H_2_O_2_ were mixed in solution and reacted for 2 h (Equation (1)), and the products were further mixed with an equivalent molar mass of GSH and reacted for another 2 h (Equation (2)).

(1) $${\mathrm{Cu}}^{+}+H_{2}O_{2}\to {\mathrm{Cu}}^{2+}+\cdot \mathrm{OH}+{\mathrm{OH}}^{-}$$

(2) $${\mathrm{Cu}}^{2+}+\mathrm{GSH}\to \mathrm{GSSG}+{\mathrm{Cu}}^{+}$$

As shown in Figure 1K, in Cu 2p XPS spectra of the oxidation products of Equation (1), the binding energy of Cu 2p_3/2_ and Cu 2p_1/2_ shifted from 952.4 and 932.6 eV to 952.7 and 932.9 eV, respectively. Meanwhile, a satellite peak of Cu (II) appeared at 944.3 eV, indicating that Cu^+^ in MC NFs was oxidized to Cu^2+^ by H_2_O_2_. In Equation (2), XPS peaks of Cu 2p_3/2_ and Cu 2p_1/2_ re‐shifted to 952.4 and 932.6 eV, respectively. Meanwhile, satellite peaks of Cu (II) also disappeared, indicating the reduction of Cu^2+^ to Cu^+^ by GSH (Figure 1L). Fourier transform infrared spectroscopy (FT‐IR) of the reduction products of Equation (2) lacked the —SH peak of GSH (≈2553 cm^−1^), like that of commercial oxidized GSH (GSSG), indicating the oxidization of —SH in GSH by Cu^2+^ (Figure 1M). Additionally, the ^1^H nuclear magnetic resonance (^1^H NMR) spectrum of the reduction product was consistent with that of commercial GSSG, further confirming GSH was oxidized to GSSG by Cu^2+^ (Figure S7, Supporting Information). The results provided strong evidence for the occurrence of cascade reactions, specifically, Cu^+^‐mediated Fenton‐like reaction and Cu^2+^‐mediated GSH depletion, which are undoubtedly favorable for continuous ROS production in tumor microenvironment (TME) with excessive H_2_O_2_ and GSH.

### 2.2 PT‐Activatable ROS Burst with MC NFs

**Figure 2:**
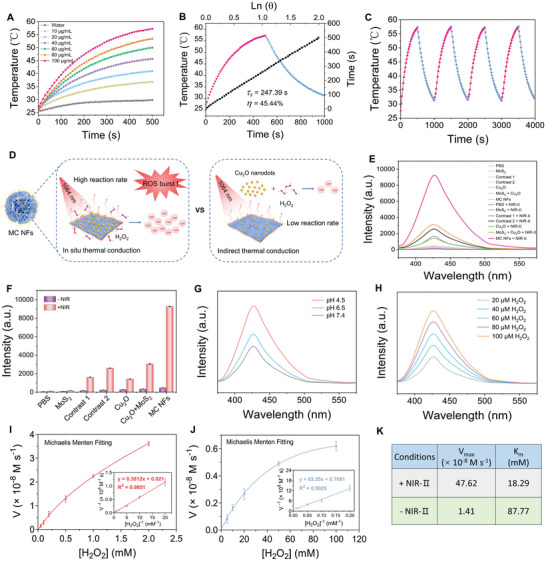
Photothermal properties and POD‐like activities of MC NFs. A) Temperature rises curves of MC NFs dispersions with different concentrations (0, 10, 20, 40, 60, 80, and 100 µg mL^−1^) under 1064 nm laser irradiation (1 W cm^−2^, 500 s). B) A temperature change of MC NFs dispersions (100 µg mL^−1^) under 1064 nm laser irradiation (1 W cm^−2^) for 500 s and followed by a cooling period (blue line). The time constant (*τ*_s_) for the heat transfer from the system was determined by applying the linear time data from the cooling period (black line). C) Heating curves of the MC NFs dispersions for four laser on/off cycles. D) Schematic illustration of MC NFs‐mediated high‐efficiency catalytic reaction. E) The generation of ·OH and F) corresponding quantitative analysis of PBS, MoS_2_, Contrast 1, Contrast 2, Cu_2_O, Cu_2_O + MoS_2_, and MC NFs with or without 1064 nm laser irradiation (1 W cm^−2^, 5 min). The ·OH generation of MC NFs at different G) pH (4.5, 6.5, and 7.4) and H) H_2_O_2_ concentrations (20, 40, 60, 80, and 100 µm). Michaelis–Menten curves and Lineweaver–Burk plotting (inset) of MC NFs I) with and J) without 1064 nm laser irradiation. K) Kinetic parameters of MC NFs with or without 1064 nm laser irradiation. Data are defined as mean ± S.D. (*n* = 3).

The PTC efficiency of the nanocomposite is one of the determinants of the generation rate of ROS.^[^ ^22^ ^]^ The MC NFs still maintain high PTC efficiency in the NIR‐II region under the condition of high‐loaded Cu_2_O (Figures S8 and S9, Supporting Information) and high ROS conversion efficiency (Figure S10, Supporting Information) by optimizing the feed ratio of Cu/Mo thanks to the advantages of copper glycinate as a precursor. As expected, MC NFs (prepared at the feed ratio of 10: 3) exhibited excellent NIR‐II absorption (Figure S11, Supporting Information), high PTC efficiency (*η* = 45.44%), and photostability (**Figure** **2**A–C). The excellent photothermal performance was also proved by infrared thermal imaging of MC NFs (Figure S12, Supporting Information). In particular, the flower‐like structure of MC NFs can allow NIR‐II light to be refracted and reflected multiple times in the inner space of MC NFs to be absorbed multiple times, which makes the PTC efficiency of MC NFs under the NIR‐II laser irradiation far exceed that of many photothermal nanomaterials, like copper phosphide nanocrystals,^[^ ^23^ ^]^ MoS_2_–CuO heterostructures,^[^ ^24^ ^]^ and black phosphorus nanosheets.^[^ ^25^ ^]^

MC NFs can generate more powerful ROS storms than many ordinary nanoplatforms under NIR‐II light irradiation because of their high PTC efficiency and high density of Cu_2_O grown in situ (Figure 2D). To further confirm our hypothesis, the ROS generation abilities of MC NFs, Contrast 1, Contrast 2, pure Cu_2_O, and the mixture of Cu_2_O and MoS_2_ were compared with terephthalic acid (TA) as the ·OH probe. Expectedly, MC NFs showed significantly higher ROS conversion efficiency relative to the other groups, the ROS fluorescence intensity of MC NFs + NIR‐II group was 21.6 times higher than that without laser irradiation (Figure S13, Supporting Information), which was much higher than that of most PT‐enhanced CDT agents, such as FeF_2_/Fe_1−_ *_x_* S nanorods,^[^ ^26^ ^]^ Pt‐decorated Ti_3_C_2_T*_x_* MXene,^[^ ^27^ ^]^ and Zn*_x_* Mn_1−_ *_x_* S@polydopamine hollow nanospheres.^[^ ^28^ ^]^ However, the catalytic of nanoenzymes may be compromised by the TME with a relatively low H_2_O_2_ concentration (50–100 µm). Encouragingly, even under a micromolar level of H_2_O_2_ (100 µm), the ROS intensity generated by MC NFs was 19.7 times higher than that without laser irradiation (Figure 2E,F), which was attributed to its high‐density Cu_2_O coating and substantial contribution of direct thermal conduction. Since the catalytic activity of enzymes was greatly affected by pH, we further analyzed the POD‐like properties of MC NFs over a range of pH values. POD‐like activity increased with decreasing pH, indicating that acidic conditions are more favorable for the Fenton‐like reaction mediated by MC NFs (Figure 2G). In addition, MC NFs‐mediated ·OH production displayed an H_2_O_2_ concentration‐ and NIR‐II exposure time‐dependent increase (Figure 2H and Figure S14, Supporting Information). To examine the POD‐like catalytic mechanism of MC NFs, steady‐state kinetics were studied using TA as ·OH probe. As shown in Figure 2I,J, the steady‐state kinetic of MC NFs was well consistent with classical Michaelis–Menten kinetics. The kinetic parameters were further calculated by the corresponding Lineweaver–Burk plot, including maximum reaction velocity (*V*_max_) and Michaelis–Menten constant (*K*_m_). As shown in Figure 2K, *V*_max_ of MC NFs was calculated as 47.62 × 10^−8^ M s^−1^ under NIR‐II laser irradiation, which was 33.8 times that of *V*_max_ (1.41 × 10^−8^ M s^−1^) in the absence of NIR‐II laser irradiation. Under NIR‐II laser irradiation, the *K*_m_ of MC‐NFs was calculated to be 18.29 mm, which was much lower than the group without NIR‐II light irradiation (87.77 mm), indicating the affinity of MC NFs for H_2_O_2_ is much higher under this condition. Therefore, the as‐prepared MC NFs have excellent photothermal performance and photothermally enhanced POD‐like activity, which can act as a NIR‐II photoactivatable “ROS bomb” to elicit a strong synergistic PTT‐CDT effect.

### 2.3 Preparation and Lysosomal Toxicity of MCPQZ

**Figure 3:**
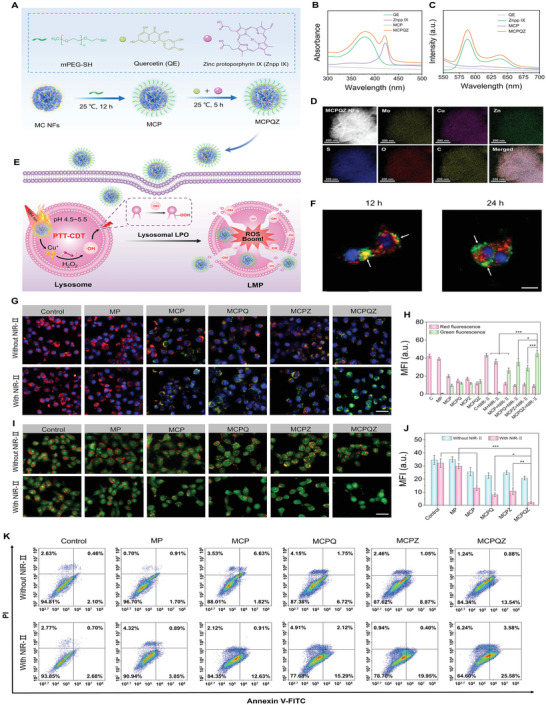
A) Schematic illustration of the synthesis of MCPQZ. B) UV–vis and C) fluorescence spectrum of QE, Znpp IX, MCP, and MCPQZ. D) Element mapping of MCPQZ. E) Schematic illustration of the MCPQZ‐induced LMP by PTT‐CDT synergistic effect. F) Co‐localization between FITC‐labeled MCPQZ and lysosome in 4T1 cells at different time points (12 and 24 h). The scale bar represents 20 µm. G) Fluorescence images and H) corresponding mean fluorescence intensity of C11‐BODIPY^581/591^‐stained 4T1 cells treated with various nanomaterials with or without 1064 nm laser irradiation (1 W cm^−2^, 5 min). The scale bar represents 50 µm. I) Fluorescence images and J) corresponding mean fluorescence intensity of AO‐stained 4T1 cells treated with various nanomaterials with or without 1064 nm laser irradiation (1 W cm^−2^, 5 min). The scale bar represents 50 µm. K) Flow cytometric analysis for apoptotic 4T1 cells treated with various nanomaterials with or without 1064 nm laser irradiation (1 W cm^−2^, 5 min). The data are presented as mean ± S.D. (*n* = 3). ^*^ *p* < 0.05, ^**^ *p* < 0.01, ^***^ *p* < 0.001 versus MCPQZ + NIR‐II.

In view of their excellent photothermal and NIR‐II activatable POD‐like properties, we expect MC NFs to have efficient anti‐tumor activity. However, cancer cells have a strong ability to reduce the therapeutic efficiency of nanodrugs through a variety of defense mechanisms. On the one hand, hyperthermia induced by PTT causes significant upregulation of heat shock proteins, which initiates refolding of damaged proteins and consequent resistance to thermal damage.^[^ ^29^ ^]^ On the other hand, innate antioxidant defense mechanisms of tumor cells are activated by elevated ROS, such as overexpression of HO‐1 that catalyzes heme to produce a series of endogenous ROS scavengers and protect against oxidative stress induced by CDT.^[^ ^30^ ^]^ To overcome these challenges, the HSP70 expression inhibitor, QE, and HO‐1 activity inhibitor, Znpp IX, were co‐loaded on PEGylated MC NFs (MoS_2_–Cu_2_O–PEG [MCP]) via electrostatic adsorption and *π*–*π* stacking to obtain a versatile MCPQZ nanoplatform (**Figure** **3**A). Subsequent TEM imaging revealed well‐formed 3D flower‐like nanostructures of MCPQZ (Figure S15A, Supporting Information) with an average hydrodynamic size of 290.67 nm (Figure S15B, Supporting Information). Following PEG modification and co‐loading with QE and Znpp IX, the zeta potentials of MCP and MCPQZ were higher than that of MC NFs (Figure S15C, Supporting Information). As shown in Figure 3B, the two peaks at around 375 nm and 420 nm in the UV–vis spectrum of MCPQZ corresponded to characteristic absorption of QE and Znpp IX, respectively. In addition, characteristic fluorescence emission peaks of Znpp IX near 595 and 640 nm were observed in the MCPQZ spectrum (Figure 3C), and XPS spectroscopy (Figure S16, Supporting Information) and element mapping further revealed distribution of Mo, S, Cu, O, C, and Zn elements on the surface of MCPQZ (Figure 3D), indicative of successful loading of QE and Znpp IX. FT‐IR spectra confirmed that methoxy polyethylene glycol mercapto (mPEG‐SH) was successfully modified onto MC NFs (Figure S17A, Supporting Information). MC NFs contained some defects at the edge to expose Mo atoms. Therefore, mPEG‐SH could be efficiently modified onto MC NFs through S—Mo bond. Besides, DLS results suggested the average hydrodynamic diameter of MCPQZ has negligible changes for 7 days of incubation in PBS and Dulbecco's modified eagle medium (DMEM) (containing 10% fetal bovine serum [FBS]), revealing the excellent system stability of MCPQZ (Figure S17B, Supporting Information). MoS_2_ nanosheet has a graphene‐like sheet structure and property. Correspondingly, MoS_2_ nanosheet has been widely reported to efficiently load drugs containing conjugated carbon structures.^[^ ^31^ ^]^ In addition, MC NFs have a unique flower‐like structure, endowing MC NFs with a huge void space to simultaneously load QE and Znpp IX. The drug loading efficiency (DLE) of QE and Znpp IX were 66.12% and 10.32% in MCPQZ, respectively. Moreover, QE and Znpp IX of MCPQZ could be efficiently released in a lysosome‐like environment (pH 4.5) (Figure S18, Supporting Information). Notably, MCPQZ could continue to release Znpp IX after 12 h, and the effect of Znpp IX could be guaranteed in the in vivo treatment experiment. Encouragingly, functionalization of PEG and co‐loading of QE and Znpp IX had no significant influence on the catalytic performance of MC NFs under NIR‐II laser irradiation, and the POD‐like catalytic behavior of MCPQZ was similar to that of MC NFs under similar conditions (Figure S19, Supporting Information).

Subsequently, rhodamine B (RhB) and fluorescein isothiocyanate (FITC) were used to label MCPQZ to verify whether MCPQZ can effectively enter 4T1 cells and its distribution in organelles, respectively (Figure S20, Supporting Information). First, we investigated the cellular uptake of RhB‐labeled MCPQZ under different incubation times (0, 6, and 12 h). The results showed that the internalized amount of RhB‐labeled MCPQZ increased with the incubation times, indicating efficient cellular uptake of MCPQZ (Figure S21, Supporting Information). Cu^+^‐mediated Fenton‐like reaction preferentially occurs in acidic lysosomes to induce lysosomal lipid peroxidation (LPO) by the cytotoxic ·OH produced under NIR‐II laser irradiation, resulting in LMP and cancer cell apoptosis (Figure 3E). As illustrated in Figure 3F, LysoTracker red‐stained lysosomes (red fluorescence) colocalized with FITC‐labeled MCPQZ (green fluorescence) to generate yellow fluorescence after co‐incubation for 12 h, suggesting that MCPQZ could be effectively captured by lysosomes. At 24 h, a clear separation of red fluorescence from lysosomes and green fluorescence from FITC‐labeled MCPQZ was observed, revealing the escape of MCPQZ from lysosomes into cytosol. Lysosomal LPO induced by MCPQZ was further investigated with C11‐BODIPY^581/591^ dye, a ratiometric fluorescent probe that shows a shift in fluorescence from red (reduced state) to green (oxidized state) in the presence of lipid hydrogen peroxide. As shown in Figure 3G,H, untreated 4T1 cells showed strong red fluorescence while other groups showed a certain degree of red fluorescence reduction and green fluorescence enhancement owing to a slight CDT effect in acidic lysosomes. Importantly, the red fluorescence of MCP, MoS_2_–Cu_2_O–PEG@QE (MCPQ), MoS_2_–Cu_2_O–PEG@Znpp IX (MCPZ), and MCPQZ groups decreased notably after NIR‐II irradiation while green fluorescence increased significantly, indicating that the photothermal effect greatly promoted intracellular oxidative stress induced by Cu^+^‐mediated CDT. Among these, the MCPQZ + NIR group exhibited the strongest green fluorescence, which was attributed to the potent lysosomal LPO elicited by MCPQZ. The lysosomal membrane integrity of 4T1 cells following different treatments was further evaluated with acridine orange (AO) staining. AO is a pigment with membrane permeability that emits green fluorescence in the cytoplasm and nucleus and red fluorescence in acidic organelles, particularly lysosomes. As shown in Figure 3I,J, green fluorescence and obvious red fluorescence were observed in the control group, indicative of intact lysosomal membrane integrity. However, red fluorescence was significantly diminished in 4T1 cells after co‐incubation with different nanomedicines for 12 h followed by NIR‐II laser irradiation, suggesting damage to the lysosomal membrane. Cells treated with MCPQZ under NIR‐II laser exposure showed no trace of red fluorescence, signifying the most severe loss of lysosomal membrane integrity. Importantly, LMP caused by loss of membrane integrity facilitates release of cathepsins in lysosomes into the cytoplasm, inducing apoptosis of cancer cells. Therefore, the major anti‐tumor mechanism of MCPQZ is speculated to involve lysosomal disruption by ·OH‐induced LPO, thus initiating LMP‐mediated lysosome‐related cancer cell death. As expected, MCPQZ induced the highest total apoptosis ratio of 4T1 cells (29.16%) with the annexin V‐FITC/PI assay, followed by MCPZ (20.35%), MCPQ (17.38%), MCP (13.54%), and MP (4.74%) (Figure 3K and Figure S22, Supporting Information).

### 2.4 ROS Burst of MCPQZ Triggered by NIR‐II Laser

**Figure 4:**
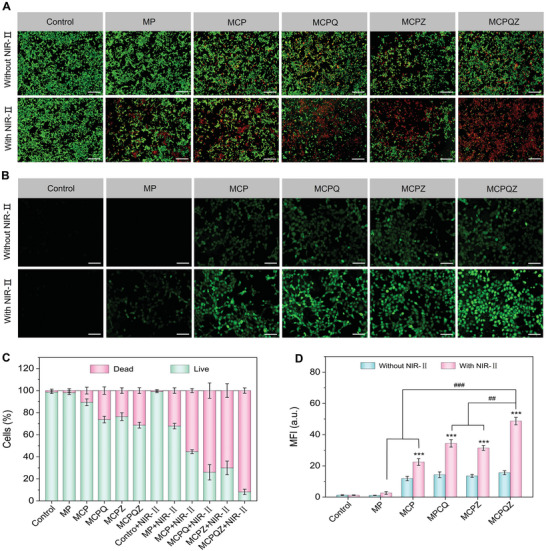
A) Fluorescence images of live/dead staining of 4T1 cells treated with different nanomaterials with/without 1064 nm laser irradiation (1 W cm^−2^, 5 min). The scale bar represents 200 µm. B) Fluorescence images of intracellular ROS detection treated with different nanomaterials with/without 1064 nm laser irradiation (1 W cm^−2^, 5 min). The scale bar represents 100 µm. C) Quantitative analysis of live/dead staining. D) Mean fluorescence intensity of intracellular ROS level in different groups. The data are presented as mean ± S.D. (*n* = 3). ^***^ *p* < 0.001 versus control + NIR‐II, ^#^ *p* < 0.05, ^##^ *p* < 0.01, ^###^ *p* < 0.001 versus MCPQZ + NIR‐II.

The PTT‐CDT synergistic anticancer effect of MCPQZ was further evaluated in vitro in view of their efficient cellular uptake and strong lysosome disruption ability. Cell counting kit‐8 (CCK‐8) assay was utilized to investigate the biocompatibility of MCPQZ on rat myocardial cells H9C2 and its anticancer effect on 4T1 cells. Although treated with a high dose of MCPQZ (200 µg mL^−1^), negligible toxicity was observed after co‐incubated with H9C2 cells, indicating the excellent biocompatibility of MCPQZ (Figure S23A, Supporting Information). As shown in Figure S23B, Supporting Information, the viability of 4T1 cells was decreased after incubation with different doses of MCPQZ in a concentration‐dependent manner. Importantly, cell viability decreased significantly after NIR‐II laser irradiation, which was attributed to the strong synergistic effect of PTT‐CDT. The cytotoxicity of MCPQZ was further elevated in comparison to other control groups (MP, MCP, MCPQ, MCPZ, and MCPQZ). As shown in Figure S23C,D, Supporting Information, cytotoxicity was greatly augmented in all therapeutic groups compared with the control group. MCPQZ induced the most significant decrease in cell viability (as low as 20.7%), followed by MCPQ (38.6%), MCPZ (40.5%), MCP (49.9%), and MP (67.1%) under NIR‐II laser irradiation. The killing effect of MCPQZ on 4T1 cells was further verified by live/dead cell staining based on the calcein‐AM/PI assay. As expected, intense red fluorescence from PI (representing dead cells) and negligible green fluorescence from calcein‐AM (representing live cells) were observed in MCPQZ‐treated 4T1 cells compared with the other therapeutic groups under NIR‐II laser irradiation (**Figure** **4**A). The proportion of dead cells in the MCPQZ + NIR‐II group was as high as 91.8%, signifying the strongest cytotoxicity (Figure 4C).

To evaluate intracellular oxidative stress induction by MCPQZ, 2,7‐dichlorodihydrofluorescein diacetate (DCFH‐DA) was adopted as a fluorescent ROS probe. As shown in Figure 4B, slight green fluorescence was observed in 4T1 cells treated with MCP, MCPQ, MCPZ and MCPQZ while negligible green fluorescence was detected in the control and MP groups, supporting the occurrence of Cu^+^‐mediated CDT in TME with high concentrations of H_2_O_2_. Compared with control and MP groups, green fluorescence was significantly enhanced in MCP‐, MCPQ‐, MCPZ‐, and MCPQZ‐treated 4T1 cells under NIR‐II laser irradiation. The mean fluorescence intensity of the MCPQZ + NIR‐II group was the highest (27.5 times higher relative to control), demonstrating the promising PTT‐CDT synergistic effect of multifunctional MCPQZ (Figure 4D). Based on these results, PTT‐induced hyperthermia greatly improved the efficiency in triggering a ROS burst to induce potent oxidative stress in 4T1 cells during MCPQZ‐mediated CDT.

### 2.5 MCPQZ Effectively Eliminating Multiple Defense Systems

**Figure 5:**
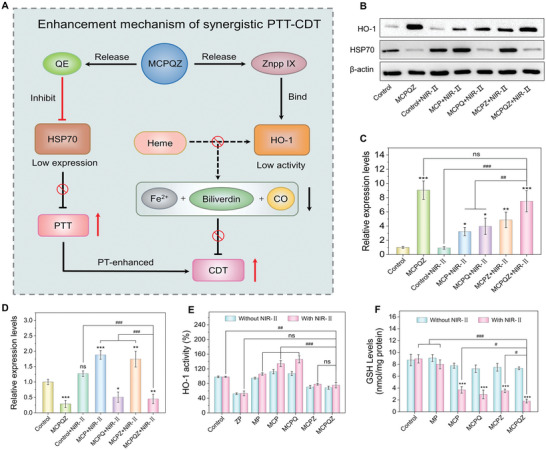
A) Enhancement mechanisms of MCPQZ‐mediated PTT‐CDT synergistic therapy. B) Western blotting analysis of HSP70 and HO‐1 expressions in 4T1 cells after different treatments and corresponding semiquantitative analysis of C) HSP70 and D) HO‐1. E) HO‐1 activity in 4T1 cells after different treatments. F) Intracellular GSH levels in different groups. The data are presented as mean ± S.D. (*n* = 3). ^*^ *p* < 0.05, ^**^ *p* < 0.01, ^***^ *p* < 0.001 versus control, ^#^ *p* < 0.05, ^##^ *p* < 0.01, ^###^ *p* < 0.001 versus MCPQZ + NIR‐II, ns: no significant difference.

In addition to MoS_2_‐mediated PTT and photothermally enhanced CDT, the anticancer activity of MCPQZ was augmented by the dual therapeutic effects of QE and Znpp IX in PTT and CDT, respectively (**Figure** **5**A). To clarify the mechanisms underlying the dual enhancement effect of QE and Znpp IX in MCPQZ‐mediated PTT‐CDT synergistic therapy, expression of HSP70 and HO‐1 in 4T1 cells subjected to different treatments were detected via western blot and activity of HO‐1 was measured with the bilirubin assay. As shown in Figure 5B,C, HSP70 levels of 4T1 cells in MCP and MCPZ treatment groups were significantly upregulated compared with the control groups after NIR‐II laser irradiation, indicating that PTT‐induced hyperthermia promoted HSP70 expression. Interestingly, QE effectively reversed the upregulation of HSP70 induced by hyperthermia, as evidenced by the significant decrease of HSP70 expression in MCPQZ, MCPQ + NIR‐II, and MCPQZ + NIR‐II groups. Conversely, HO‐1 was upregulated to varying degrees in all treatment groups. Both MCPQZ and MCPQZ + NIR‐II groups showed a significant increase in HO‐1 expression (Figure 5B,D). Notably, HO‐1 activity was markedly inhibited by Znpp IX, MCPZ, and MCPQZ in 4T1 cells, while other treatment groups without Znpp IX displayed enhanced HO‐1 activity compared to the control group (Figure 5E). A reasonable explanation for these results is that high HO‐1 expression is mediated by the NF‐E2‐related factor‐2 signaling pathway in response to enhanced cellular oxidative stress injury. Therefore, significant upregulation of HO‐1 in MCPQZ‐treated 4T1 cells indirectly confirmed the strong ability of MCPQZ to induce intracellular oxidative stress. The antioxidant activity of HO‐1 is proposed to originate from the regiospecific catabolism of heme to produce endogenous scavengers of ROS. Znpp IX released from MCPQZ prevented the decomposition of heme and subsequent production of antioxidants by competitively binding the active site of HO‐1, but had no effect on the abundance of HO‐1 in 4T1 cells.

Intracellular GSH levels were additionally measured using the 5,5‐dithio‐bis‐(2‐nitrobenzoic acid) (DTNB) method. As shown in Figure 5F, MCPQZ caused greater GSH depletion than other therapeutic groups under NIR‐II irradiation (4.9 and 4.4 times higher relative to control and MP groups, respectively), indicating significantly reduced clearance of intracellular ROS and an enhanced CDT effect of this nanocomposite. Taken together, these results suggested that simultaneous inhibition of HSP70 expression and HO‐1 activity greatly weakens the antioxidant defense ability of cancer cells, thus significantly enhancing the synergistic effect of PTT‐CDT respectively.

### 2.6 MCPQZ for Treatment of 4T1 Tumor‐Bearing Mice

**Figure 6:**
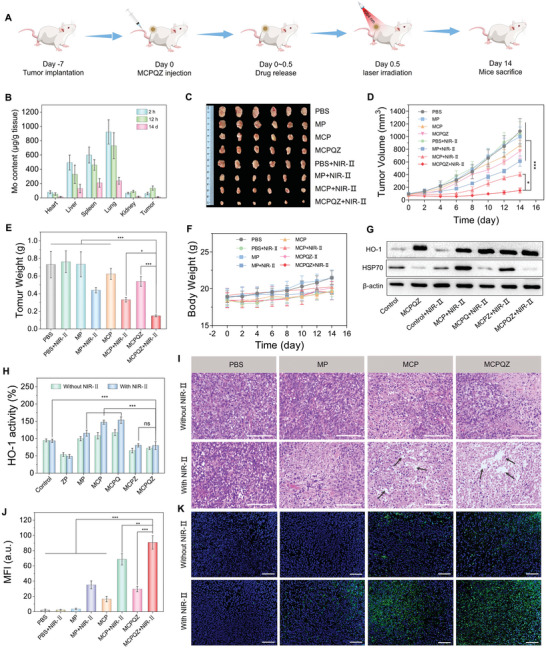
A) Scheme of the anti‐tumor experiment by MCPQZ‐mediated PTT‐CDT synergistic therapy. B) Biodistribution of MCPQZ in main organs and tumors at different time points. C) The representative photos of tumors dissected at 14th day post‐treatment. D) Tumor growth curves of mice after various treatments for 14 days. E) Tumor weight at the end of the treatment. F) The body weight changes of 4T1 tumor‐bearing mice in the treatment process. G) Western blotting analysis of HSP70 and HO‐1 expressions in tumor tissues after different treatments. H) HO‐1 activity in tumor tissues after different treatments. I) H&E‐stained photographs of tumor slices obtained from tumor‐bearing mice treated with various treatments after 14 days. The scale bar represents 100 µm. K) TUNEL‐stained photographs of tumor slices obtained from tumor‐bearing mice treated with various treatments after 14 days and J) corresponding mean fluorescence intensity. The scale bar represents 50 µm. The data presented as mean ± S.D. (*n* = 6). ^*^ *p* < 0.05, ^**^ *p* < 0.01, ^***^ *p* < 0.001 versus MCPQZ + NIR‐II.

Considering the efficient anti‐tumor activity of MCPQZ in vitro, 4T1 tumor‐bearing mice were used to investigate the MCPQZ‐mediated PTT‐CDT synergistic therapy in vivo (**Figure** **6**A). After intravenous administration, the Mo content in main organs and tumor tissues of 4T1 tumor‐bearing mice was analyzed via inductively coupled plasma mass spectrometry (ICP‐MS). The results of biodistribution of MCPQZ showed the effective accumulation in tumor tissues (Figure 6B). The mononuclear phagocyte system has a high distribution in the lung, liver, and spleen, where macrophages engulf foreign nanoparticles (as size increases beyond 150 nm).^[^ ^32^ ^]^ As a respiratory organ, the lung frequently exchanges substances with the external environment, so it has highly rich resident macrophages. Correspondingly, a part of MCPQZ was resident in the lungs after intravenous injection. Fortunately, MCPQZ can be effectively cleared by the lung within 14 days owing to its good degradability, thereby avoiding potential systemic toxicity caused by long‐term retention. In addition, accumulation of MCPQZ was the highest in tumor tissues at 12 h after administration (Figure S24, Supporting Information). Subsequently, 4T1 tumor‐bearing mice were treated with 1064 nm laser irradiation (1 W cm^−2^, 5 min) at 12 h post‐administration of MCPQZ. As shown in Figure 6C–E, MCPQZ + NIR‐II exerted the most pronounced tumor growth inhibition effect, followed by MCP + NIR‐II, MP + NIR‐II, MCPQZ, MCP, and MP in order. In addition, the weight fluctuations of mice in all groups were negligible, which preliminarily confirmed the in vivo biosafety of these MoS_2_‐based nanomaterials (Figure 6F). As expected, tumor tissue expression of HSP70 and activity of HO‐1 showed a similar trend to in vitro results, further validating the dual enhancement effect of MCPQZ‐mediated PTT‐CDT synergistic therapy (Figure 6G,H and Figure S25, Supporting Information). Hematoxylin and eosin (H&E) staining of tumor tissues showed that MCPQZ caused the most severe tumor lesions under NIR‐II laser irradiation relative to other therapeutic groups, as evident from the condensed nuclei of tumor cells and enlarged tissue space (black arrow, Figure 6I). Terminal deoxynucleotidyl transferase‐mediated dUTP nick end labeling (TUNEL) staining confirmed that the MCPQZ + NIR‐II group also induced more significant apoptosis of tumor tissues than other groups (Figure 6J,K).

Owing to the high spatiotemporal selectivity of PTT‐CDT synergistic therapy, no obvious pathological changes were observed with H&E staining of the skin tissues surrounding the tumor in MCPQZ + NIR‐II group compared with the control group, indicating the high biosafety of MCPQZ‐mediated PTT‐CDT (Figure S26, Supporting Information). Meanwhile, the negligible pathological changes of H&E staining of main organs after treatment with MCPQZ suggested no significant adverse effects in vivo. Additionally, blood indexes, including parameters related to blood panel counts and blood biochemistry of mice treated with MCPQZ, were not significantly different compared with the control group, further validating the excellent in vivo biocompatibility of MCPQZ (Figures S27 and S28, Supporting Information). In conclusion, MCPQZ can efficiently accumulate in tumor tissues and achieve a remarkable PTT‐CDT‐mediated synergistic anti‐tumor effect combined with excellent biosafety in vivo, supporting its potential utility as a photothermal‐enhanced nanocatalyst for high‐efficiency synergistic cancer therapy.

## 3 Conclusion

In summary, a NIR‐II photoactivatable “ROS bomb” was developed to tackle the key bottleneck of ROS‐based therapy. The as‐prepared MC NFs exhibited efficient NIR‐II absorption, considerable PTC efficiency (45.44%), and hyperthermia‐amplified POD‐like activity due to the unique flower‐like structure of MoS_2_ and high‐density Cu_2_O coating. Under NIR‐II laser irradiation, the *V*_max_ of MC NFs‐mediated Fenton‐like reaction was 33.8 times that of the group without NIR‐II laser irradiation in vitro, which was much higher than that of most current nanocatalysts. QE and Znpp IX were co‐loaded on PEG‐modified MC NFs to construct a multifunctional MCPQZ nanoplatform. A facile and high‐efficiency sequential catalytic therapy is as follows: First, QE and Znpp IX pre‐release from MCPQZ to overcome thermal resistance and antioxidant defense of cancer cells by downregulating the expression of HSP70 and activity of HO‐1, respectively, building a favorable microenvironment for subsequent thermal and ROS injury. Second, under a 1064 nm laser irradiation, CDT effect of MCPQZ can be greatly enhanced by PTT‐induced hyperthermia, generating a potent ROS storm via Cu (I)‐catalyzed Fenton‐like reaction and Cu (II)‐mediated GSH depletion (ROS intensity increased by 27.8 times compared to the control).

The development of highly effective anticancer drugs remains a highly promising and is an unmet need. The key to the prospect of anticancer drugs lies in therapeutic effect, toxic side effects, and biocompatibility. MCPQZ has a high therapeutic effect on cancer, and can greatly avoid toxic side effects in the treatment of cancer thanks to the excellent spatiotemporal selectivity of NIR‐II laser therapy. Moreover, both Mo and Cu in MCPQZ are the essential trace elements for human survival. Many Mo‐containing enzymes (such as xanthine oxidase, aldehyde oxidase) and copper‐containing enzymes (superoxide dismutase, cytochrome C oxidase) existing in human body play an important role in human metabolism. Overall, this work provides novel insights to develop more efficient and safer nanocatalytic therapy for future clinical applications.

## 4 Experimental Section

### Materials and Reagents

Ammonium molybdate tetrahydrate ((NH_4_)_6_Mo_7_O_24_·4H_2_O, AR, 99%), thiourea (CN_2_H_4_S, ≥99%), copper glycinate (C_4_H_8_CuN_2_O_4_), QE (C_15_H_10_O_7_, 97%), disodium terephthalate (C_8_H_4_Na_2_O_4_), and AO (C_17_H_20_ClN_3_·HCl·1/2ZnCl_2_) were purchased from Macklin. Zinc protoporphyrin IX (C_34_H_32_N_4_O_4_Zn, >95%) was purchased from Beijing Bailingwei Technology Co., Ltd. Methoxy polyethylene glycol mercapto (mPEG‐SH, MW 5000) was obtained from Shanghai Yarebio Co., Ltd. FITC isomer I was obtained from Aladdin. DMEM and FBS were purchased from Thermo Fisher Scientific. CCK‐8, 2,7‐dichlorodihydrofluorescein diacetate (DCFH‐DA), Hoechst 33342, and LysoTracker Red were purchased from Shanghai Beyotime Biotechnology. Live–dead cell staining kit was obtained from Shanghai Maokang Biotechnology Co., Ltd. C11‐BODIPY^581/591^ dye was purchased from Abclonal. Annexin V‐FITC/PI apoptosis detection kit was purchased from Dalian Meilun Biotechnology Co., Ltd.

### Synthesis of MoS_2_ Nanoflowers

MC NFs were synthesized by the hydrothermal method. Specifically, (NH_4_)_6_Mo_7_O_24_⋅4H_2_O (18.5375 g) and CN_2_H_4_S (7.9925 g) were dissolved in 300 mL deionized water under vigorous stirring for 30 min. The above mixture was then transferred to a 300 mL Teflon‐lined stainless steel autoclave and kept at 180 °C for 24 h. After cooling to room temperature, the products were centrifuged at 12 000 rpm for 10 min and repeated washing with ultrapure water three times, and eventually, the MC NFs were obtained after drying at 60 °C under vacuum overnight. In addition, PEG‐modified MC NFs were synthesized for comparison. The prepared MC NFs (50 mg) were dispersed in 50 mL of deionized water, and then mPEG‐SH (50 mg) was added into the above solution. After stirring at room temperature for 12 h, repeated washing with ultrapure water was done to remove free mPEG‐SH to obtain PEGylated MoS_2_ (MoS_2_–PEG, MP).

### Synthesis of MoS_2_–Cu_2_O Nanoflowers, Contrast 1, and Contrast 2

MoS_2_–Cu_2_O NFs were further synthesized by the second hydrothermal process. MC NFs with different content of Cu by varying the feed ratio (i.e., the molar mass ratio of Mo and Cu [*n* [Mo]:*n* [Cu] = 10:1, 10:2, 10:3, 10:4, and 10:5]) were prepared. Taking MC NFs prepared at a feed ratio of 10:3 as an example, the prepared MC NFs (60 mg) were dispersed in 40 mL ultrapure water, then 23.8 mg of copper glycinate was added into the above solution under vigorous stirring for 30 min. The above mixture was transferred to a 50 mL Teflon‐lined stainless‐steel autoclave and kept at 180 °C for 8 h. After cooling to room temperature, the products were collected through centrifugation at 12 000 rpm for 10 min, and washed with ultrapure water for several times, and eventually the MC NFs were obtained after drying at 60 °C under vacuum overnight. Similarly, the Contrast 1 was prepared by the second hydrothermal process using MC NF as the substrate and copper chloride as the Cu precursor. The Contrast 2 was prepared by a one‐step hydrothermal method using ammonium molybdate tetrahydrate, thiourea, and copper chloride as the Mo, S, and Cu precursor, respectively.

### Synthesis of MoS_2_–Cu_2_O–PEG@QE/Znpp IX

First, The PEGylated MoS_2_–Cu_2_O (MoS_2_–Cu_2_O–PEG, MCP) were obtained by the above method. Then, 1 mL QE (15 mg mL^−1^ in DMSO) and 1 mL Znpp IX (3 mg mL^−1^ in DMSO) were added into 12 mL MCP NFs (15 mg) dispersions and stirred at room temperature for 5 h. After that, the products were centrifuged at 12 000 rpm for 15 min, the supernatants were collected, and the DLE of QE and Znpp IX were calculated according to the absorption of QE at *λ* = 375 nm and Znpp IX at *λ* = 420 nm, respectively. The DLE was calculated by the Equation (3):

(3) $$\mathrm{DLE}\;\left(\%\right)=\frac{\mathrm{Initial}\;\mathrm{Wt}.\;\mathrm{of}\;\mathrm{drug}\;-\;\mathrm{Wt}.\;\mathrm{of}\;\mathrm{drug}\;\mathrm{in}\;\mathrm{supernatant}}{\mathrm{Initial}\;\mathrm{Wt}.\;\mathrm{of}\;\mathrm{drug}}$$

Subsequently, the products were centrifuged and washed with ultrapure water several times to obtain MCPQZ. According to the DLE of MCPQZ, MCPQ, and MCPZ were prepared as described above. Briefly, 0.8 mL QE (15 mg mL^−1^ in DMSO) or 0.1 mL Znpp IX (3 mg mL^−1^ in DMSO) were added into 15 mg MCP and made the total volume to 15 mL with deionized water. After stirring at room temperature for 5 h, the products were centrifuged and washed with ultrapure water several times to obtain MCPQ or MCPZ.

### Preparation of RhB‐Labeled MCPQZ and FITC‐Labeled MCPQZ

The prepared MCPQZ (15 mg) were dispersed in 14 mL deionized water, then 1 mL RhB or FITC (5 mg mL^−1^ in DMSO) was added and stirred at room temperature for 5 h in the dark. After that, the products were centrifuged and washed with ultrapure water for several times. Finally, the products were dispersed in ultrapure water and dialyzed (MWCO = 3500) for 24 h to obtain RhB‐labeled MCPQZ or FITC‐labeled MCPQZ.

### Physicochemical Characterization

TEM system (JEOL JEM‐2100F) was used to investigate the morphology and microstructure of the synthesized materials. Zeta potential and hydrodynamic size were determined by Malvern instrument (Zetasizer Nano). The elemental composition was analyzed by TEM mapping. XPS spectra were measured through a VG ESCALAB 250Xi X‐ray photoelectron spectrometer. XRD patterns were obtained using a Smartlab SE X‐ray diffractometer (Rigaku, Japan). ICP‐OES (ICAP‐6300) was used to determine the content of Mo and Cu elements in the synthesized materials. FT‐IR spectra were measured using a Perkin Elmer spectrometer. UV–vis absorption spectra were observed by a TU‐1810DSPC spectrophotometer (Persee, Beijing, China). Fluorescence spectrum was recorded by F98 fluorescence spectrometer (Lengguang Technology, Shanghai, China).

### MC NFs‐Mediated Redox Reaction

10 mL MC NFs suspension (50 mm) and 10 mL H_2_O_2_ (50 mm) were mixed and reacted at room temperature for 2 h. After the reaction was completed, the supernatant was centrifuged at 12 000 rpm for 10 min and washed with ultra‐pure water three times to obtain the oxidation product of MC NFs. The oxidation product was dispersed in 10 mL ultra‐pure water and mixed with 10 mL GSH (50 mm) for 2 h, then centrifuged at 12 000 rpm for 10 min. The supernatant was collected and washed for three times with ultra‐pure water to obtain the reduction product of MC NFs. The valence of Cu in the oxidation product and reduction product was determined by XPS. In addition, FT‐IR and ^1^H NMR were used to prove the generation of GSSG in the reduction product.

### Photothermal Properties of MC NFs

First, in order to investigate the photothermal effect of MC NFs prepared by different molar mass ratio (*n* [Mo]:*n* [Cu]) in raw materials, 1064 nm laser with a power density of 1 W cm^−2^ was used to irradiate MC NFs suspensions (100 µg mL^−1^) for 500 s and followed by cooling for 500 s naturally, and the temperature change of the suspensions was monitored with a digital probe thermometer (Invesible, T‐105) every 10 s. The PTC efficiency (*η*) was calculated according to the previous reports. The *η* was calculated by Equation (4):

(4) $$\eta \;=\frac{hS\left(T_{\max}-T_{\mathrm{surr}}\right)-Qs}{I\left(1-10^{-A_{\lambda }}\right)}$$

where *η* was the PTC efficiency, *h* was the heat transfer coefficient of the container (W m^−2^ °C^−1^), *S* was the surface area of the container (m^2^), *T*_max_ was the maximum temperature reached by the system, *T*_surr_ was the ambient temperature, and *Qs* was the power absorption of the solvent under 1064 nm laser irradiation (W), *I* was the laser power density (W cm^−2^), and *A_*λ*_* was the absorbance of the MC NFs at 1064 nm. The value of *hS* was calculated by Equation (5):

(5) $$\tau_{s}=\frac{m_{D}c_{D}}{hS}$$

where *τ*_s_ was the time constant of the sample system, *m*_D_ was the mass of water, and *c*_D_ was the heat capacity of water (4.2 J g^−1^ °C^−1^).

According to the calculation results of PTC efficiency, when *n* (Mo):*n* (Cu) = 10: 3, the photothermal efficiency of MC NFs was the highest, so the MC NFs with the feed ratio at 10:3 was selected as the follow‐up research object. First, UV–vis spectrophotometer was used to detect the near‐infrared absorption properties of MC NFs suspensions with different concentrations (20, 40, 60, 80, and 100 µg mL^−1^) at 700–1100 nm. The extinction coefficient (*ε*) of the MC NFs was determined by the Lambert–Beer Law:

(6) $$A_{\lambda }=\varepsilon CL$$

where *A_*λ*_* was the optical density at the wavelength (*λ*) of 1064 nm, *L* (cm) was the length of the laser beam in the absorbing medium, and *C* (g L^−1^) was the concentration of the MC NFs. Finally, *ε* was calculated by plotting the slope of each linear fit against wavelength. Then, photothermal heating curves of MC NFs with different concentrations (20, 40, 60, 80, and 100 µg mL^−1^) under 1064 nm laser irradiation (1 W cm^−2^, 5 min) were measured by the digital probe thermometer. In addition, the photothermal responsive performance of MC NFs also verified by infrared thermal imaging (Arrow Eye‐T2). Afterward, four laser on/off cycles were used to evaluate the photothermal stability of MC NFs.

### POD‐Like Activity of MC NFs and MCPQZ

Disodium terephthalate (TA) could react with ·OH to form a stable fluorescent substance 2‐hydroxyterephthalic acid (TA‐OH). The ·OH generation of MC NFs and MCPQZ were evaluated by the fluorescence intensity of TA‐OH at 430 nm. First of all, the ·OH generation of five kinds of MC NFs with different copper content were investigated with or without NIR‐II laser irradiation. Specifically, MC NFs (final concentration 50 µg mL^−1^) were dispersed in 1.5 mL PBS solution (0.01 m, pH 4.5), and then 100 µL TA solution (final concentration 50 mm) and 400 µL H_2_O_2_ solution (final concentration 100 mm) were added. Then, a 1064 nm laser with a power density of 1 W cm^−2^ was used to continuously irradiate the mixed system for 5 min, a F98 fluorescence spectrometer was used to detect the fluorescence intensity at 430 nm to evaluate the generation of ·OH. Then, the ratio of fluorescence intensity (*F*_+NIR‐II_/*F*_−NIR‐II_) at 430 nm with/without NIR‐II laser irradiation was calculated, which represented the contribution of NIR‐II laser to the ·OH generation of MC NFs. According to the calculated *F*_+NIR‐II_/*F*_−NIR‐II_ value, the MC NFs with the feeding ratio at 10:3 was selected as the follow‐up research object.

First, to simulate the real situation in the TME, the ·OH generation of MC NFs, Contrast 1, Contrast 2, pure Cu_2_O, and the mixture of Cu_2_O and MoS_2_ were investigated under the NIR‐II laser irradiation or not. In short, MC NFs, Contrast 1, Contrast 2, pure Cu_2_O, and the mixture of Cu_2_O and MoS_2_ containing the same amount of Cu_2_O (30 µg) were dispersed in PBS solution (0.01 m, pH 4.5). After adding 100 µL TA (final concentration 50 µm) and 400 µL H_2_O_2_ (final concentration 100 µm), the mixed solutions were incubated for 5 min with or without NIR‐II laser, respectively. Then the fluorescence intensity at 430 nm was detected immediately by F98 fluorescence spectrometer (Voltage parameter: high gain). In addition, the ·OH generation of MC NFs (final concentration 50 µg mL^−1^) under different pH (7.4, 6.5, and 4.5), H_2_O_2_ concentrations (20, 40, 60, 80, and 100 µm), and NIR‐II laser exposure time (0.5, 1, 2, 3, 4, and 5 min) was also explored. Similarly, the above results were also measured at a mm level H_2_O_2_ concentration (100 mm) by F98 fluorescence spectrometer (Voltage parameter: medium gain). Also, the ·OH generation of MCPQZ was further measured to explore the influence of PEG modification, QE, and Znpp IX loading on the POD‐like activity of MCPQZ.

### Kinetic Assay of MC NFs

First of all, the standard concentration curve of 2‐hydroxyterephthalic acid (TA‐OH) at low concentration was determined for follow‐up fluorescence quantitative analysis. Subsequently, the steady‐state kinetic assays were performed in PBS solution (2 mL, 0.01 m, pH 4.5) with MC NFs (final concentration 50 µg mL^−1^) in various tubes. Then, TA solution (final concentration 2.5 mm) and various concentrations of H_2_O_2_ were added to above mixture, followed by a 1064 nm laser irradiation or not. Finally, the fluorescence intensity at the 430 nm was detected immediately (within the linear range of the standard concentration curve of TA‐OH). According to the fluorescence intensity, the corresponding concentration was calculated by the standard concentration curve of TA‐OH, then the reaction rate was determined according to the value of the concentration, and the Michaelis–Menten saturation curve was obtained by curve fitting. The kinetic parameters, including the *V*_max_ and the *K*_m_ were determined by Michaelis–Menten (Equation (7)) and Lineweaver–Burk (Equation (8)),

(7) $$\;V_{0}=\frac{V_{\max}\;\left[S\right]}{K_{m}+\left[S\right]}$$

(8) $$\frac{1}{V_{0}}=\frac{K_{m}\;}{V_{\max}}\;\cdot \frac{1}{\left[S\right]}+\frac{1}{V_{\max}}$$

### In Vitro Drug Release

The release of QE and Znpp IX was measured by a modified method according to the previous study. For QE release, MCPQZ dispersions containing 1 mg QE were dispersed in PBS buffer (pH = 4.5, 7.4) containing 2% Tween 80, and then incubated in a thermostatic shaker (37 °C, 150 rpm). At the predetermined time points, the sample aliquots were collected and the absorbance of QE was determined by UV–vis spectrophotometer at *λ*_max_ of 375 nm, and the cumulative release of the QE was recorded periodically according to the standard concentration working curve. For Znpp IX release, MCPQZ NFs containing 0.5 mg Znpp IX were dispersed in PBS buffer (pH = 4.5, 7.4) containing 0.5% Tween 80, and then incubated in a thermostatic shaker (37 °C, 150 rpm). Sample aliquots were collected at pre‐set time points and the absorbance of Znpp IX was measured by UV–vis spectrophotometer at *λ*_max_ of 420 nm, and the cumulative release of the Znpp IX was recorded periodically according to the standard concentration working curve.

### Cellular Uptake and ·OH‐Induced Lysosomal Membrane Permeabilization

*Cellular uptake*: 4T1 cells were seeded in 12‐well plates (1 × 10^5^ cells per well) and incubated overnight. To investigate the cellular internalization of MCPQZ, RhB‐labeled MCPQZ (equivalent to 100 µg mL^−1^ MoS_2_ or 85.96 µg mL^−1^ QE or 2.69 µg mL^−1^ Znpp IX) were incubated with 4T1 cells for 6 or 12 h. After that, 4T1 cells were washed with PBS three times, and stained with Hoechst 33342 for 20 min. Fluorescence images were then acquired with Zeiss inverted fluorescence microscope (Axio vert. A1.). Untreated cells served as a control group. Furthermore, to investigate the subcellular localization of MCPQZ, FITC‐labeled MCPQZ were incubated with 4T1 cells in an FBS‐free medium (equivalent to 100 µg mL^−1^ MoS_2_ or 85.96 µg mL^−1^ QE or 2.69 µg mL^−1^ Znpp IX) for 12 or 24 h. The lysosome and nuclei were stained with LysoTracker red (50 nm, 30 min) and Hoechst 33342 (20 min), respectively, then intracellular localization of MCPQZ was observed by fluorescence microscope.

*·OH‐induced lysosomal membrane permeabilization*: C11‐BODIPY^581/591^ dye and AO were used to evaluate lipid peroxidation and lysosomal membrane permeabilization in 4T1 cells after different nanomaterials treatments, respectively. Specifically, 4T1 cells were seeded in 24‐well plates (5 × 10^4^ cells per well) and incubated overnight. 4T1 cells were co‐cultured with MP, MCP, MCPQ, MCPZ, and MCPQZ (equivalent to 100 µg mL^−1^ MoS_2_ or 85.96 µg mL^−1^ QE or 2.69 µg mL^−1^ Znpp IX) for 12 h. Untreated cells served as a control group. After washed with PBS three times and replaced with fresh medium, 4T1 cells were exposed to a 1064 nm laser (1 W cm^−2^, 5 min) or not, and incubated for another 2 h. After washed three times with PBS, 4T1 cells were stained with C11‐BODIPY^581/591^ dye (10 µm) or AO (5 µm) for 30 min, followed by Hoechst 33342 for 20 min to indicate cell nuclei before observation with fluorescence microscope.

### In Vitro Anti‐Tumor Effect

The biocompatibility of MCPQZ was first tested by CCK‐8 assay. Briefly, H9C2 myocardial cells were seeded in 96‐well plates (1 × 10^4^ cells per well) and incubated overnight. Then, the cells were incubated with MCPQZ with different concentrations (0, 6.125, 12.5, 25, 50, 100, and 200 µg mL^−1^) for 24 h. For CCK‐8 assay, each well was added 100 µL of CCK‐8 solution and incubated for another 1 h, and the absorbance at 450 nm was recorded by a DTX880 multifunctional microplate reader.

*In vitro anti‐tumor effect of MCPQZ*: 4T1 cells were seeded in 96‐well plates (1 × 10^4^ cells per well) and incubated overnight. Then, the cells were incubated with MCPQZ with different concentrations (0, 6.125, 12.5, 25, 50, 100, and 200 µg mL^−1^) for 12 h. After changing normal culture medium, the cells were irradiated with a 1064 nm laser (1 W cm^−2^) for 5 min or not, and incubated for another 12 h. Untreated cells served as a control group. The cell viability was evaluated by CCK‐8 assay described above. In addition, the viability of 4T1 cells treated with MP, MCP, MCPQ, MCPZ, and MCPQZ (equivalent to 100 µg mL^−1^ MoS_2_ or 85.96 µg mL^−1^ QE or 2.69 µg mL^−1^ Znpp IX) with/without 1064 nm laser irradiation was also evaluated by the CCK‐8 assay. For the live/dead staining, after treatment with MP, MCP, MCPQ, MCPZ, and MCPQZ (equivalent to 100 µg mL^−1^ MoS_2_ or 85.96 µg mL^−1^ QE or 2.69 µg mL^−1^ Znpp IX) for 12 h and changing the normal culture medium, the cells were irradiated with a 1064 nm laser (1 W cm^−2^) for 5 min or not, and incubated for another 12 h. Then, the cells were washed with PBS three times and stained with calcein‐AM (2 µm) and PI (4 µm) for 30 min. After washing three times with PBS, the fluorescence images were captured using fluorescence microscope.

### Intracellular ROS Level Detection

DCFH‐DA was employed as a fluorescent ROS probe to indicate oxidative stress induced by MCPQZ. Briefly, 4T1 cells were seeded in 24‐well plates (5 × 10^4^ cells per well) and incubated overnight. Then, the cells were treated with MP, MCP, MCPQ, MCPZ, and MCPQZ (equivalent to 100 µg mL^−1^ MoS_2_ or 85.96 µg mL^−1^ QE or 2.69 µg mL^−1^ Znpp IX) for 12 h. Untreated cells served as a control group. After replacing with fresh medium, the cells were irradiated with a 1064 nm laser (1 W cm^−2^) for 5 min or not, and incubated for another 2 h. After washed with PBS three times, the cells were stained with DCFH‐DA (10 µm) for 30 min, and the fluorescence images were captured.

### Intracellular GSH Level Detection

GSH level was measured by DTNB method. 4T1 cells were seeded in 24‐well plates (5 × 10^4^ cells per well) and incubated overnight. Then, the cells treated with MP, MCP, MCPQ, MCPZ, and MCPQZ (equivalent to 100 µg mL^−1^ MoS_2_ or 85.96 µg mL^−1^ QE or 2.69 µg mL^−1^ Znpp IX) for 12 h. After replacing with fresh medium, the cells were irradiated with a 1064 nm laser (1 W cm^−2^) for 5 min or not, and incubated for another 12 h. Untreated cells served as a control group. After washed with PBS three times, the cells were scraped and lysed with pre‐cooled RIPA lysis buffer (P0013B, Beyotime, China), then the cell lysates were centrifuged at 12 000 rpm for 15 min at 4 °C. After that, the supernatant was collected and the protein concentration was determined by the BCA method (P0010, Beyotime, China), and 30 µL of supernatant was mixed with 150 µL of DTNB (30 µg mL^−1^) for 30 min, and the absorbance at 412 nm was measured by UV–vis spectrophotometer. Different concentrations of GSH solutions (0, 20, 40, 60, 80, and 100 µm) were used as standard for quantitative analysis of GSH level.

### Cellular HSP70 and HO‐1 Expression

The expression of HSP70 and HO‐1 in 4T1 cells was analyzed by western blotting. Specifically, 4T1 cells were seeded in 12‐well plates (1 × 10^5^ cells per well) and incubated overnight. Then, the cells were treated with MCP, MCPQ, MCPZ, and MCPQZ (equivalent to 100 µg mL^−1^ MoS_2_ or 85.96 µg mL^−1^ QE or 2.69 µg mL^−1^ Znpp IX) for 12 h. After replacing the fresh medium, the cells were irradiated with a 1064 nm laser (1 W cm^−2^) for 5 min or not, and incubated for another 12 h. Untreated cells served as a control group. The cells were scraped and lysed with pre‐cooled RIPA lysis buffer (P0013B, Beyotime, China), then the cell lysates were centrifuged at 12 000 rpm for 15 min at 4 °C. After that, the supernatant was collected and the protein concentration was determined by the BCA method (P0010, Beyotime, China). The supernatant was fixed with loading buffer and then denatured at 99 °C for 7 min, and the obtained protein was stored at −20 °C until use. 20 µg of total protein from each sample was separated by 10% sodium dodecyl sulfate‐polyacrylamide gel electrophoresis electrophoresis assay and transferred to polyvinylidene fluoride membranes. The membranes were blocked with 5% skim milk in TBST buffer (pH 7.4, 0.1% Tween‐20) for 1.5 h at room temperature with constant shaking, followed by three washes with TBST. Subsequently, the membranes were incubated with targeted primary antibodies overnight at 4 °C. After washing with TBST three times, the membranes were incubated with secondary antibodies for 1 h at room temperature. Finally, the target protein bands were imaged by a high‐sensitivity chemiluminescence imaging system (BIO‐RAD) with hypersensitive chemiluminescent (BeyoECL Plus, Beyotime, China).

### Cellular HO‐1 Activity Assay

The HO‐1 activity was evaluated by bilirubin assay. Specifically, 4T1 cells were seeded in 12‐well plates (1 × 10^5^ cells per well) and incubated overnight. 4T1 cells were then co‐incubated with ZP, MP, MCP, MCPQ, MCPZ, and MCPQZ (equivalent to 100 µg mL^−1^ MoS_2_ or 85.96 µg mL^−1^ QE or 2.69 µg mL^−1^ Znpp IX) for 12 h, after changing the fresh medium, the cells irradiated with/without 1064 nm laser (1 W cm^−2^) for 5 min, and then incubated for another 12 h. Untreated cells served as a control group. After washing three times with PBS, 1 mL of potassium phosphate buffer (0.1 m) containing 2 mm MgCl_2_ (M813765, Macklin) and protease inhibitor cocktail (B14001, Bimake) was added, and the cells were collected by gently scraping on ice, and allowed to stand for 15 min. The cells were then disrupted by ultrasonic for 15 s, 5 min/time, three times in total. Sucrose solution was added to the cell lysate to a final concentration of 0.25 m. The solution was then centrifuged at 1000 × *g* for 10 min at 4 °C, the supernatant was collected, centrifuged at 12 000 × *g* for 15 min, the supernatant was ultracentrifuged at 105 000 × *g* for 1 h, the supernatant was discarded, and the precipitate (HO‐1 crude extract) in 500 µL of potassium phosphate buffer (0.1 m) and stored at −20 °C for later use. The total protein concentration was determined by the BCA method. Then, 600 µg of crude HO‐1 extract with 400 µL of G‐glucose‐6 containing *β*‐NADPH (1 mm) (C103029, Aladdin), D‐glucose‐6‐phosphate disodium salt (2 mm) (G810516, Macklin), phosphate dehydrogenase (1 U) (G822621, Macklin), hemin (25 µm) (H886057, Macklin), and 2 mg rat liver cytoplasmic protein in potassium phosphate buffer (0.1 m) were mixed and incubated at 37 °C in the dark 1 h. The reaction mixture was then placed on ice to stop the reaction. Bilirubin concentration was determined by measuring the difference in absorbance between 465 and 530 nm.

### Cell Apoptosis Analysis

Cancer cell apoptosis was evaluated by flow cytometry based on annexin V‐FITC/PI assay. 4T1 cells were seeded in 12‐well plates (1 × 10^5^ cells per well) and incubated overnight. 4T1 cells treated with MP, MCP, MCPQ, MCPZ, and MCPQZ (equivalent to 100 µg mL^−1^ MoS_2_ or 85.96 µg mL^−1^ QE or 2.69 µg mL^−1^ Znpp IX) for 12 h. After replacing with fresh medium, the cells were irradiated with a 1064 nm laser (1 W cm^−2^) for 5 min or not, and incubated for another 12 h. Untreated cells served as a control group. Then, the cells were harvested using trypsin without EDTA, washed with PBS three times, and stained with annexin V‐FITC/PI for 15 min, followed by flow cytometry to identify the apoptotic cells.

### Tumor Xenograft

Female BALB/c mice (6–8 weeks old, purchased from Center for Experimental Animals, Central South University) were selected for establishing the xenograft mouse model. Specifically, 5 × 10^6^ 4T1 cells in 100 µL of PBS were subcutaneously injected into the right back of each mouse. All animal studies were performed in Center for Experimental Animals, Central South University, and the procedures involving experimental animals were in accordance with protocols approved by the Committee for Animal Research of Central South University, China.

### Tumor Accumulation of MCPQZ

After the 4T1 tumor‐bearing BALB/c mice model was established according to the above protocol, when the tumor reached a uniform size of around 100 mm^3^, the mice were intravenously administered with 100 µL of PBS or MCPQZ (equivalent to 10 mg MoS_2_ or 8.596 mg QE or 0.269 mg Znpp IX kg^−1^ mice). Then, the tumor tissues were dissected from mice at different time points (2, 4, 6, 8, 10, 12, 16, 20, and 24 h), and the samples were lyophilized with a freeze dryer and ground into powder. Mo content in tissues was measured by ICP‐MS to evaluate the tumor accumulation of MCPQZ in vivo (*n* = 6).

### In Vivo Anti‐Tumor Experiment and Histological Analysis

After the 4T1 tumor‐bearing BALB/c mice model was established according to the above protocol, when the tumor reached a uniform size of around 100 mm^3^, the mice were randomly divided into eight groups (*n* = 6): (1) PBS, (2) MP, (3) MCP, (4) MCPQZ, (5) PBS + NIR‐II, (6) MP + NIR‐II, (7) MCP + NIR‐II, and (8) MCPQZ + NIR‐II. Mice were intravenously injected with 100 µL of PBS or nanomaterials (equivalent to 10 mg MoS_2_ or 8.596 mg QE or 0.269 mg Znpp IX kg^−1^ mice). At 12 h post‐injection, the mice were irradiated with a 1064 nm laser (1 W cm^−2^, 5 min) or not. The tumor volume and body weight were monitored every 2 days with a digital caliper, and tumor volume was calculated by the following formula: Width^2^ × Length/2. The mice were sacrificed after 14 days of treatments, and the tumor tissues, skin tissues surrounding tumor, and major organs (heart, liver, spleen, lung, and kidney) were collected, fixed in 4% polyformaldehyde, and then embedded in paraffin, sliced and stained with H&E. For apoptosis analysis of the cancer cells, tumor tissue sections were stained for TUNEL and visualized by imaged fluorescence microscopy.

### HSP70 and HO‐1 Expression in 4T1 Tumors

After the 4T1 tumor‐bearing BALB/c mice model was established according to the above protocol, when the tumor reached a uniform size of 100 mm^3^, the mice were intravenously administered 100 µL of PBS or MCP, MCPQ, MCPZ, and MCPQZ (equivalent to 10 mg MoS_2_ or 8.596 mg QE or 0.269 mg Znpp IX kg^−1^ mice). At 12 h post‐injection, the mice were irradiated with a 1064 nm laser (1 W cm^−2^, 5 min) or not. Then, the mice were sacrificed at 12 h post‐irradiation, and the tumor tissues were collected, and the expressions of HSP70 and HO‐1 in tumor tissues were detected by western blotting as previously described.

### HO‐1 Activity in 4T1 Tumors

After the 4T1 tumor‐bearing BALB/c mice model was established, when the tumor reached 100 mm^3^, the mice were intravenously administered 100 µL of PBS or ZP, MP, MCP, MCPQ, MCPZ, and MCPQZ (equivalent to 10 mg MoS_2_ or 8.596 mg QE or 0.269 mg Znpp IX kg^−1^ mice). At 12 h post‐injection, the mice were irradiated with a 1064 nm laser (1 W cm^−2^, 5 min) or not. Then, the mice were sacrificed at 12 h post‐irradiation, and the tumor tissues were collected, and the HO‐1 activity in tumor tissues was detected by bilirubin assay as previously described.

### In Vivo Biodistribution and Safety Analysis

After the 4T1 tumor‐bearing BALB/c mice model was established, when the tumor reached to 100 mm^3^, the mice were intravenously administered 100 µL of MCPQZ (equivalent to 10 mg MoS_2_ or 8.596 mg QE or 0.269 mg Znpp IX kg^−1^ mice). The mice were sacrificed at different time points (2, 12 h, and 14 days), then the tumor tissues and major organs (heart, liver, spleen, lung, and kidney) were collected, and the samples were lyophilized with a freeze dryer and ground into powder. Mo content in tissues was measured by ICP‐MS to evaluate the in vivo biodistribution of MCPQZ (*n* = 6). For biosafety analysis of MCPQZ in vivo, after the treatment for 14 days, the blood samples were collected for complete blood panel analysis and blood biochemistry test.

### Statistical Analysis

All quantitative values were presented as mean ± standard deviation. The statistical comparison between the two groups was conducted using a two‐tailed Student's *t*‐test, and multi‐group comparisons were carried out by using ordinary one‐way ANOVA analysis followed by Tukey's post hoc test by the GraphPad Prism 8.0.1. *p* < 0.05 was considered to be statistically significant.

## Conflict of Interest

The authors declare no conflict of interest.

## Author Contributions

J.H. and G.D. contributed equally to this work. K.A. and Q.H. conceived the idea and designed the study. J.H. and G.D. conducted the experiments with the help of S.W., T.Z., and Q.C. Y.Y. and Y.Y. assisted in preparing the manuscript. J.Z. performed the data curation. K.A. and Q.H. supervised and administrated the research.

## Supporting information

Supporting Information

## Data Availability

The data that support the findings of this study are available from the corresponding author upon reasonable request.
