# Supplementary material for: A NIR‐II Photoactivatable “ROS Bomb” with High‐Density Cu2O‐Supported MoS2 Nanoflowers for Anticancer Therapy
Source: Adv Sci (Weinh). 2023 Jun 20;10(24):2302208. doi: 10.1002/advs.202302208 (PMC10460899; doi:10.1002/advs.202302208)
Supplement: Supplementary file 1 — Supporting Information [file ADVS-10-2302208-s001.pdf]

ADVANCED SCIENCE
Open Access

Supporting Information

for *Adv. Sci.*, DOI 10.1002/advs.202302208

# A NIR-II Photoactivatable "ROS Bomb" with High-Density $Cu_2O$-Supported $MoS_2$ Nanoflowers for Anticancer Therapy

*Jia Huang, Guiming Deng, Shuya Wang, Tianjiao Zhao, Qiaohui Chen, Yuqi Yang, Yongqi Yang, Jinping Zhang, Yayun Nan, Zhaoqian Liu, Ke Cao, Qiong Huang* and Kelong Ai**

# Supporting Information:

# A NIR-II Photoactivatable "ROS Bomb" with High-density $Cu_2O$-supported $MoS_2$ Nanoflowers for Anticancer Therapy

Jia Huang[1,2,3#], Guiming Deng[4#], Shuya Wang[2,3], Tianjiao Zhao[2,3], Qiaohui Chen[2,3], Yuqi Yang[1,5], Yongqi Yang[2,3], Jinping Zhang[1,5], Yayun, Nan[6], Zhaoqian Liu[1,2], Ke Cao[7], Qiong Huang[1,5*], Kelong Ai[2,3*]

[1] Department of Pharmacy, Xiangya Hospital, Central South University, Changsha, 410008, China

[2] Xiangya School of Pharmaceutical Sciences, Central South University, Changsha, 410078, China

[3] Hunan Provincial Key Laboratory of Cardiovascular Research, Xiangya School of Pharmaceutical Sciences, Central South University, Changsha, 410078, China

[4] Department of infection and liver disease, The First Hospital of Hunan University of Chinese Medicine, Changsha, 410007, China.

[5] National Clinical Research Center for Geriatric Disorders, Xiangya Hospital, Central South University, Changsha, 410008, China

[6] Geriatric Medical Center, People's Hospital of Ningxia Hui Autonomous Region, Yinchuan, Ningxia, 750002, China

[7] Department of Oncology, The Third Xiangya Hospital of Central South University, Changsha, 410013, China

[#] These authors contributed equally.

***Corresponding authors**: qionghuang@csu.edu.cn (Prof. Qiong Huang), aikelong@csu.edu.cn (Prof. Kelong Ai)

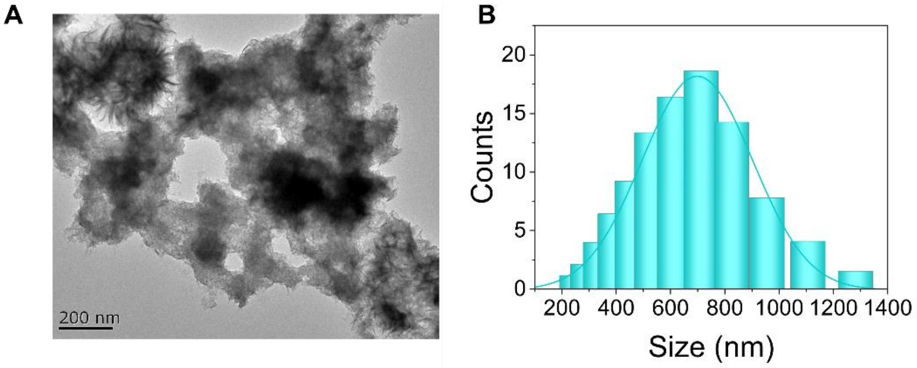


**Figure S1.** (A) TEM image and (B) Size distribution of Contrast 1.

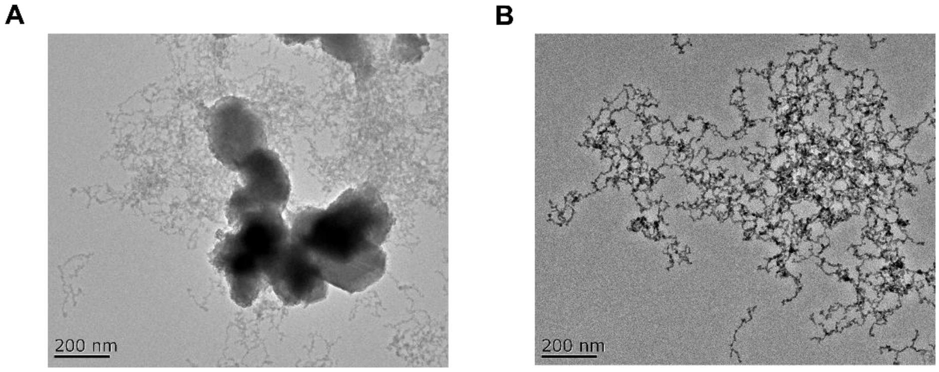


**Figure S2.** (A-B) TEM images of Contrast 2.

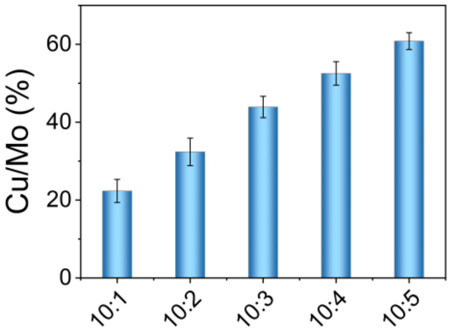


**Figure S3.** (A) The content ratio of Cu and Mo elements of MC NFs prepared at different feed ratios. Data are presented as mean ± S.D. (n = 3).

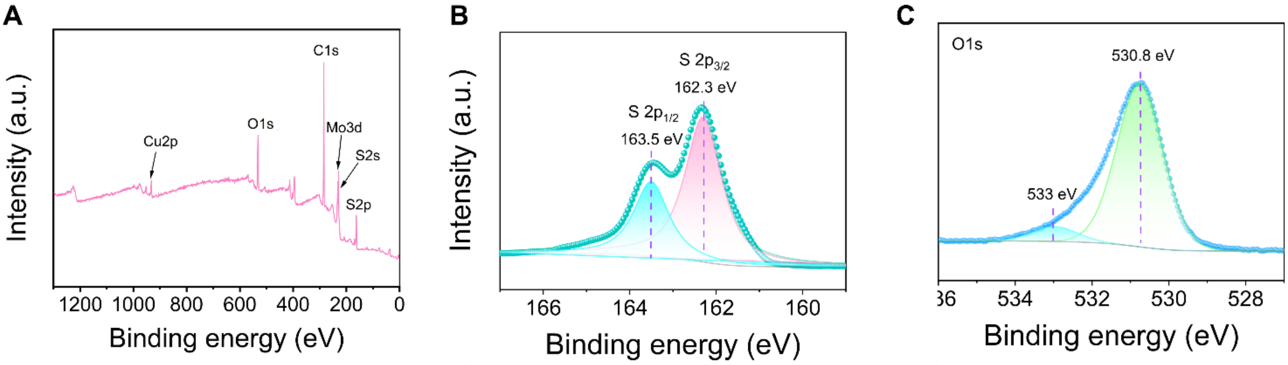


**Figure S4.** (A) XPS spectrum of MC NFs and the high-resolution of S 2p (B) and O1s (C) XPS

spectra of MC NFs.

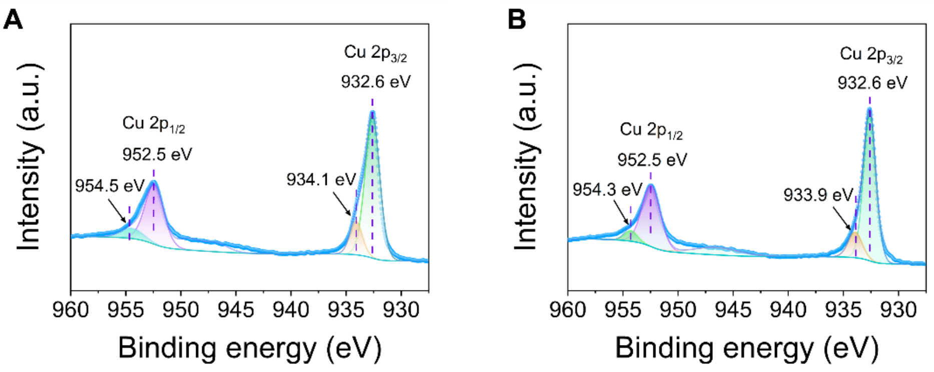


**Figure S5.** The high-resolution Cu 2p XPS spectra of (A) Contrast 1 and (B) Contrast 2.

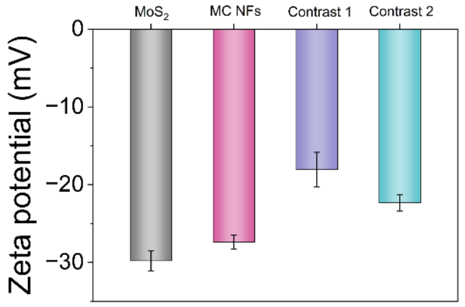


**Figure S6.** Zeta potential of $MoS_2$, MC NFs, Contras 1 and Contrast 2. Data are presented as mean $\pm$S.D. (n = 3).

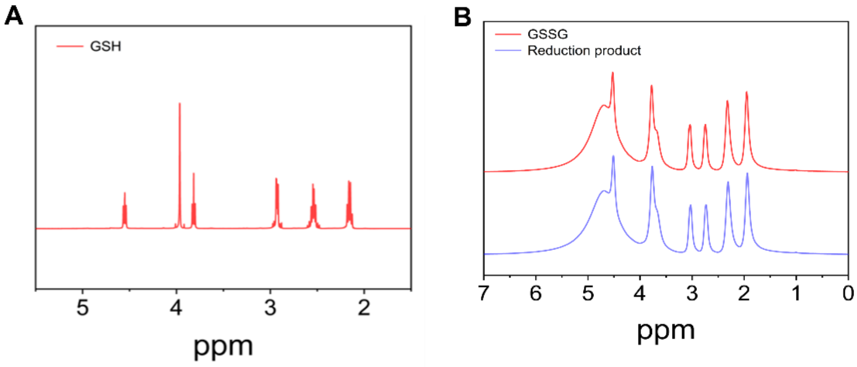


**Figure S7.** (A) $^1$H NMR spectra of GSH. (B) $^1$H NMR spectra of and commercial GSSG and the reduction product.

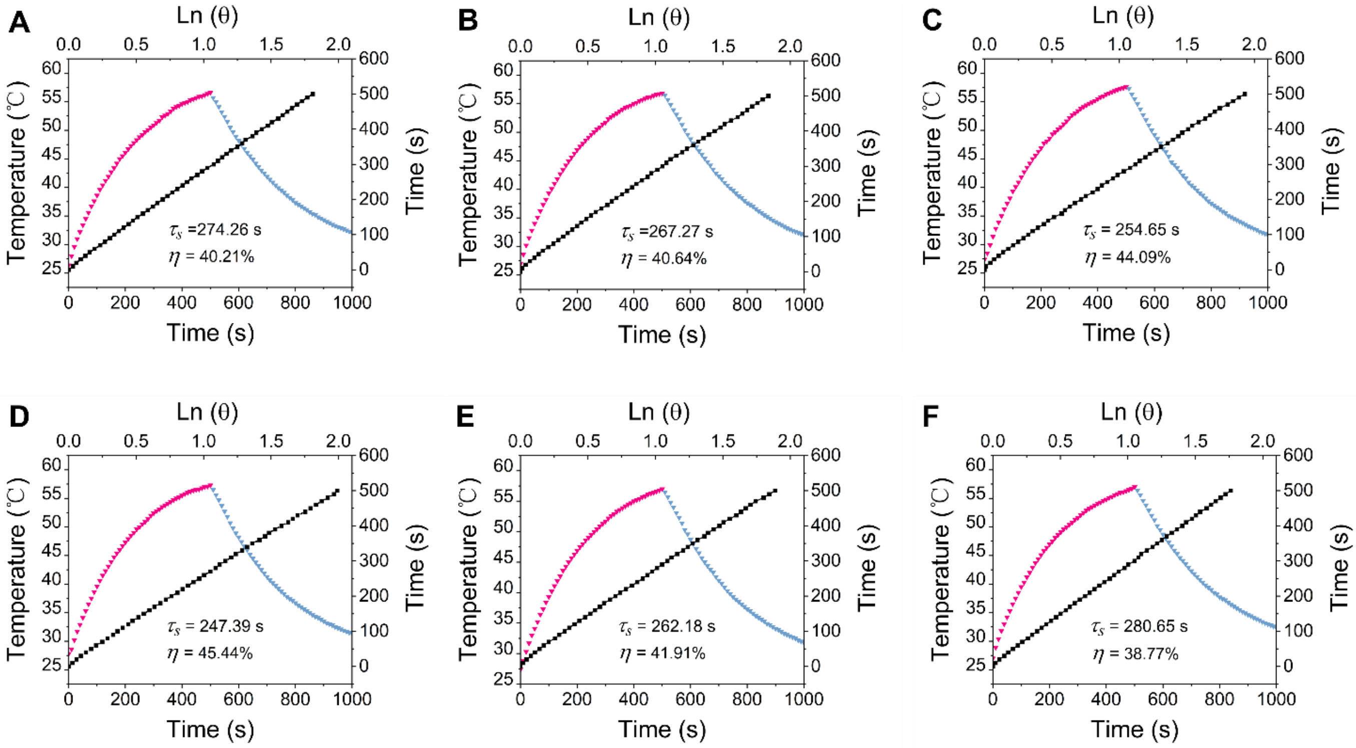


**Figure S8.** Heating and cooling curves (red and blue line) and the time constant (τs) for the heat transfer from the system were determined by applying the linear time data from the cooling period (black line) of (A) $MoS_2$, MC NFs prepared under feed ratio at (B) 10:1, (C) 10:2, (D) 10:3, (E) 10:4, and (F) 10:5.

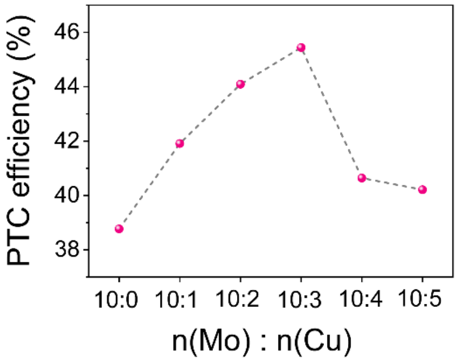


**Figure S9.** PTC efficiency of MC NFs prepared by different feed ratios.

A

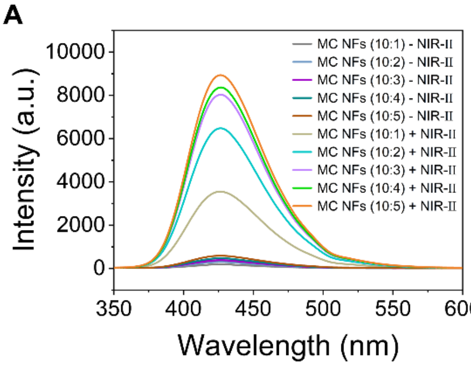


B

| Ratios | $F_{+\,NIR\text{-}II}/F_{-\,NIR\text{-}II}$ |
|---|---|
| 10:1 | 12.21 |
| 10:2 | 16.8 |
| 10:3 | 21.03 |
| 10:4 | 17.78 |
| 10:5 | 15.21 |

**Figure S10.** (A)·OH generation of MC NFs prepared with different feed ratios under 1064 nm laser irradiation or not. (B) The ratios of fluorescence intensity at 430 nm of MC NFs prepared with different feed ratios after and before 1064 nm laser irradiation ($F_{+\,NIR\text{-}II}/F_{-\,NIR\text{-}II}$).

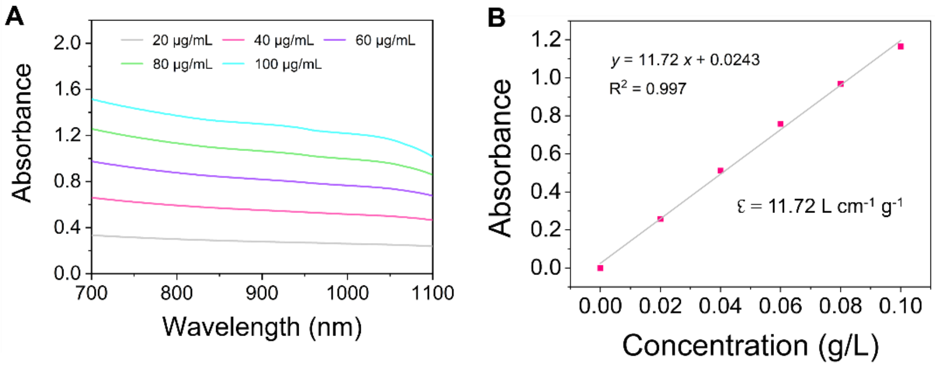


**Figure S11.** (A) UV-Vis absorption of MC NFs with different concentrations. (B) Standard absorption curves of MC NFs with different concentrations at 1064 nm.

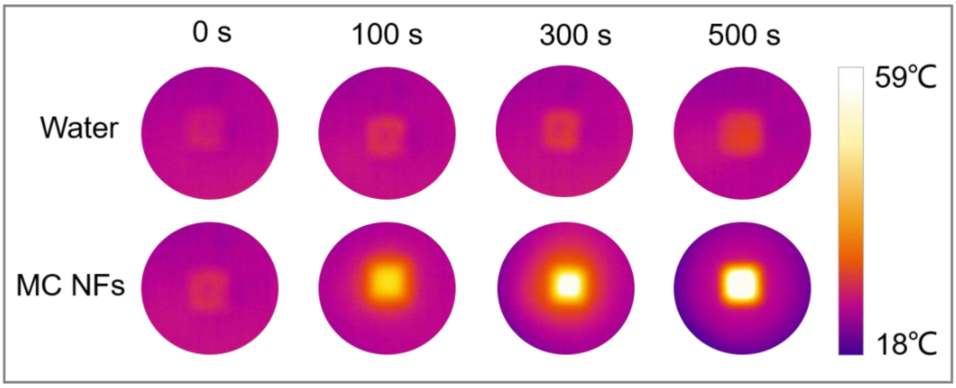


**Figure S12.** Infrared thermal imaging of MC NFs irradiated with 1064 nm laser (1 W/cm$^2$) for different times.

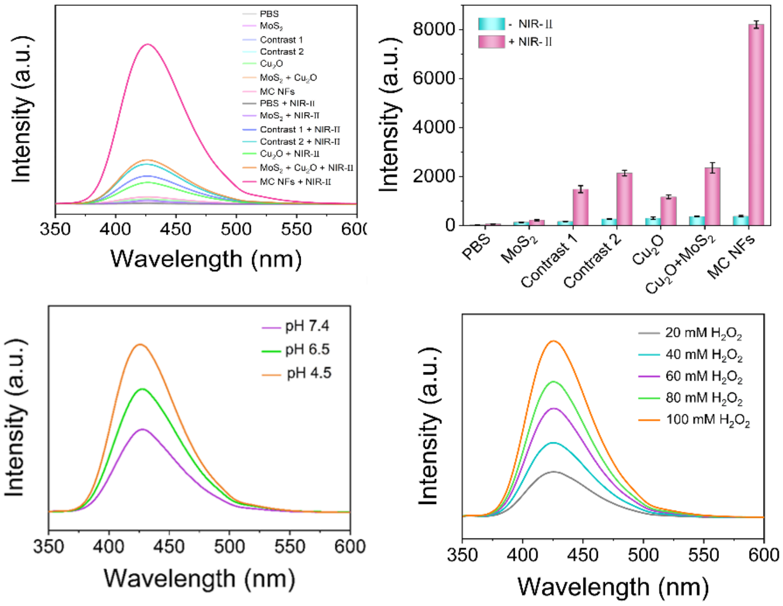


**Figure S13.** (A) The generation of ·OH and (B) corresponding quantitative analysis of PBS, $MoS_2$,

Contrast 1, Contrast 2, $Cu_2O$, $Cu_2O$ + $MoS_2$ and MC NFs at 100 mM $H_2O_2$ with 1064 nm laser irradiation (1 W cm$^{-2}$, 5 min) or not. (C) The generation of ·OH of MC NFs at different pH (4.5, 6.5, and 7.4) with fixed $H_2O_2$ concentrations (100 mM). (D) The generation of ·OH of MC NFs at different $H_2O_2$ concentrations (20, 40, 60, 80, and 100 mM) with fixed pH value (pH 4.5).

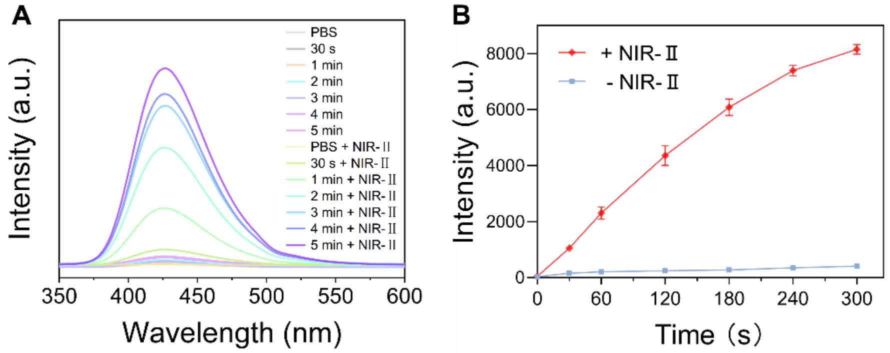


**Figure S14.** (A) The generation of ·OH and (B) corresponding statistical analysis of fluorescence intensity at different time points (0.5, 1, 2, 3, 4, 5 min) with/without 1064 nm laser irradiation (1 W cm$^{-2}$). Data are presented as mean ± S.D. (n=3).

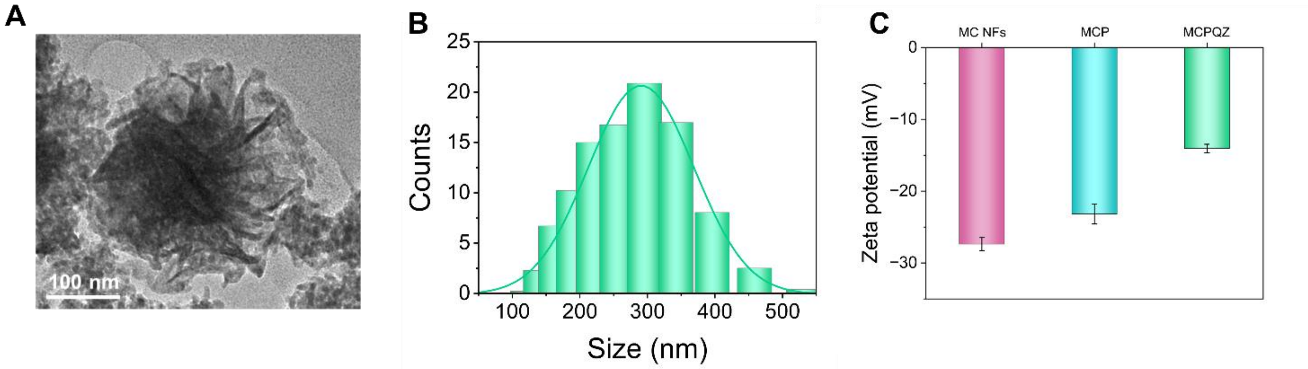


**Figure S15.** (A) TEM image and (B) size distribution of MCPQZ. (C) Zeta potential of MC NFs, MCP and MCPQZ. Data are presented as mean ± S.D. (n=3).

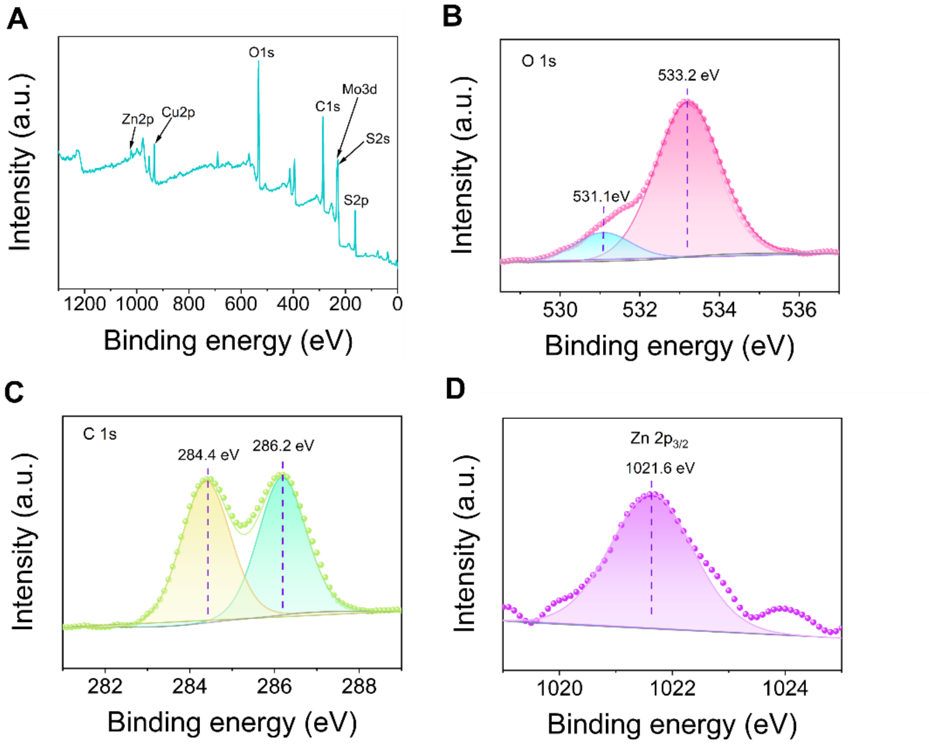


**Figure S16.** (A) XPS spectrum of MCPQZ. The high-resolution (B) O1s, (C) C 1s, and (D) Zn $2p_{3/2}$ XPS spectra of MCPQZ.

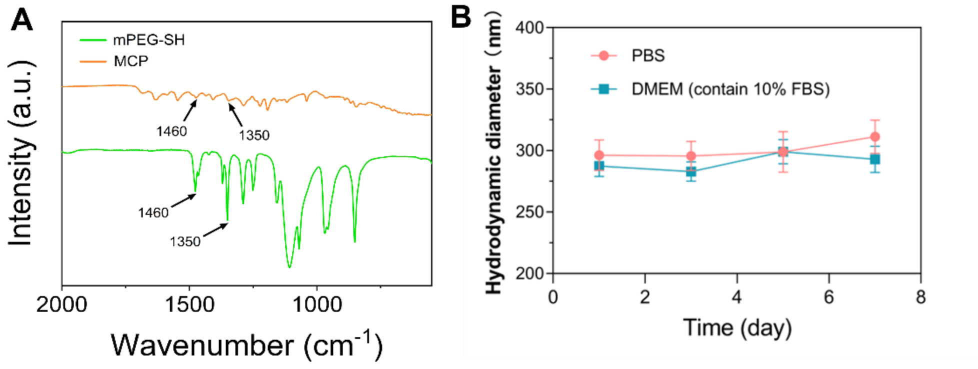


**Figure S17.** (A) FT-IR spectra of mPEG-SH and MCP. (B) The hydrodynamic diameter changes of MCPQZ measured in PBS and DMEM (contain 10% FBS). Data are presented as mean ± S.D. (n=3).

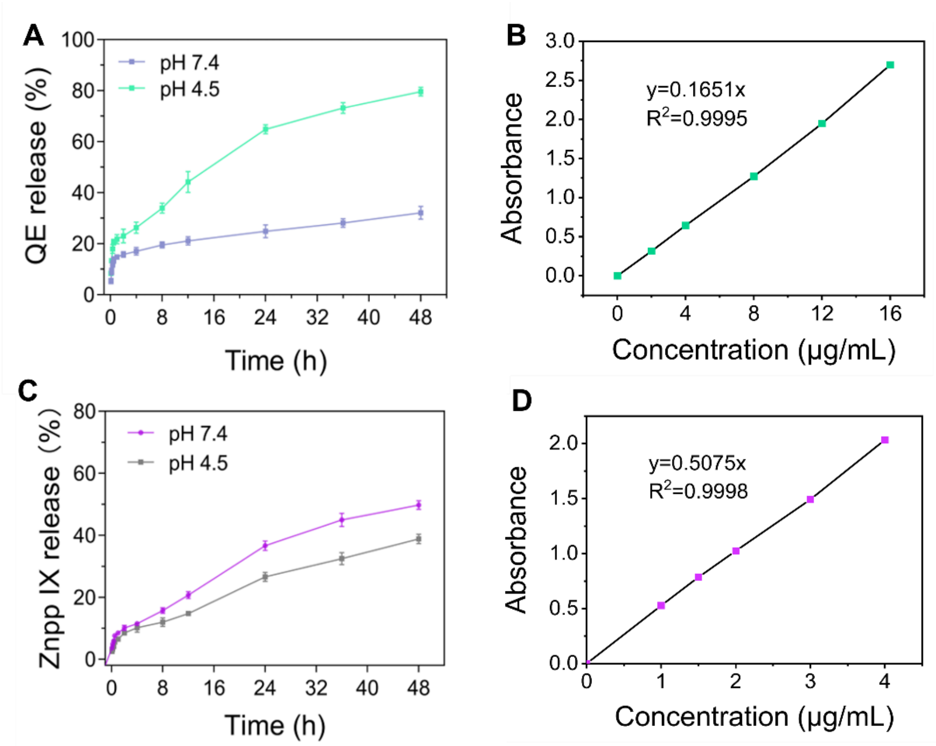


**Figure S18.** Release of QE and Znpp IX form MCPQZ. (A) QE release curves under different pH. (B) Standard concentration working curve of QE solutions. (C) Znpp IX release curves under different pH. (D) Standard concentration working curve of Znpp IX solutions.

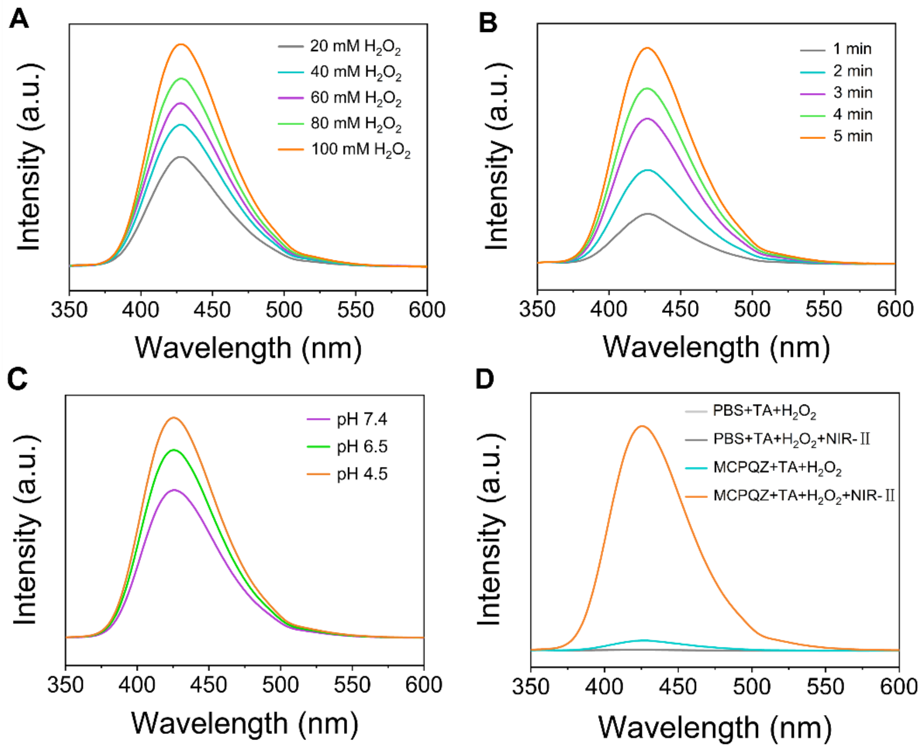


**Figure S19.** ·OH generation of MCPQZ at different (A) $H_2O_2$ concentrations (20, 40, 60, 80 and 100 μM), (B) time points (1, 2, 3, 4, 5 min) and (C) pH (4.5, 6.5 and 7.4). (D) ·OH generation of MCPQZ with/without 1064 nm laser irradiation (1 W cm$^{-2}$, 5 min).

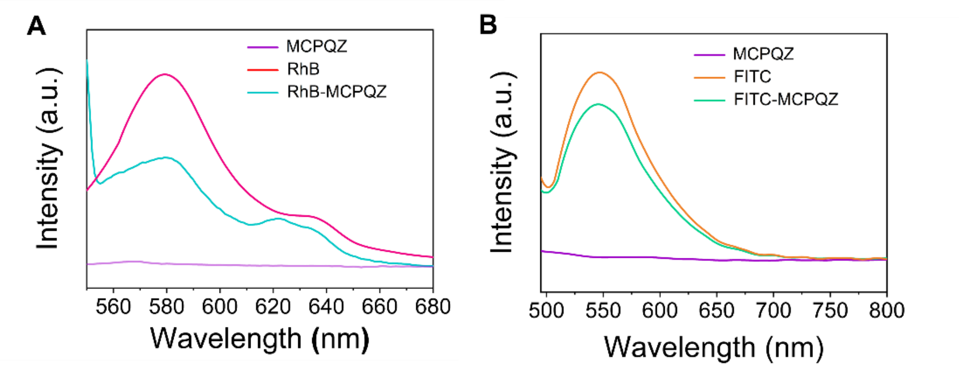


**Figure S20.** (A) Fluorescence spectrum of RhB, MCPQZ and RhB-labeled MCPQZ. (B) Fluorescence spectrum of FITC, MCPQZ and FITC-labeled MCPQZ.

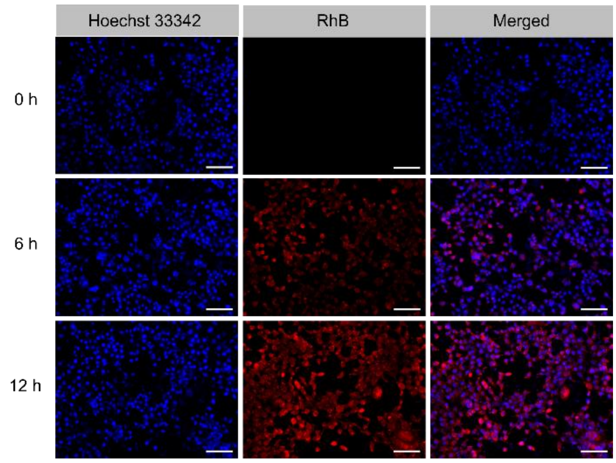


**Figure S21.** Cellular uptake of RhB-labeled MCPQZ in 4T1 cells at different time points. Scale bar = 100 μm.

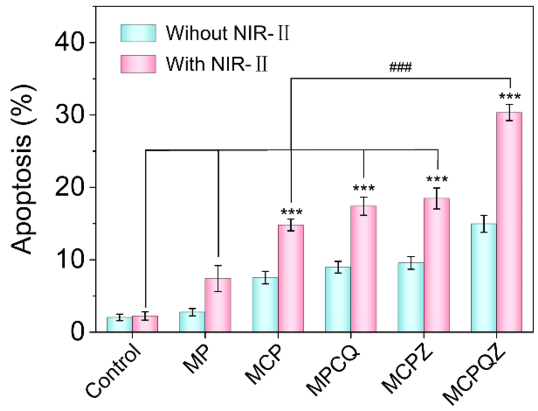


**Figure S22.** Apoptosis ratios of 4T1 cells treated with different nanomaterials with or without 1064 nm laser irradiation (1 W/cm$^2$, 5min). The data are presented as mean ± S.D. (n=3).

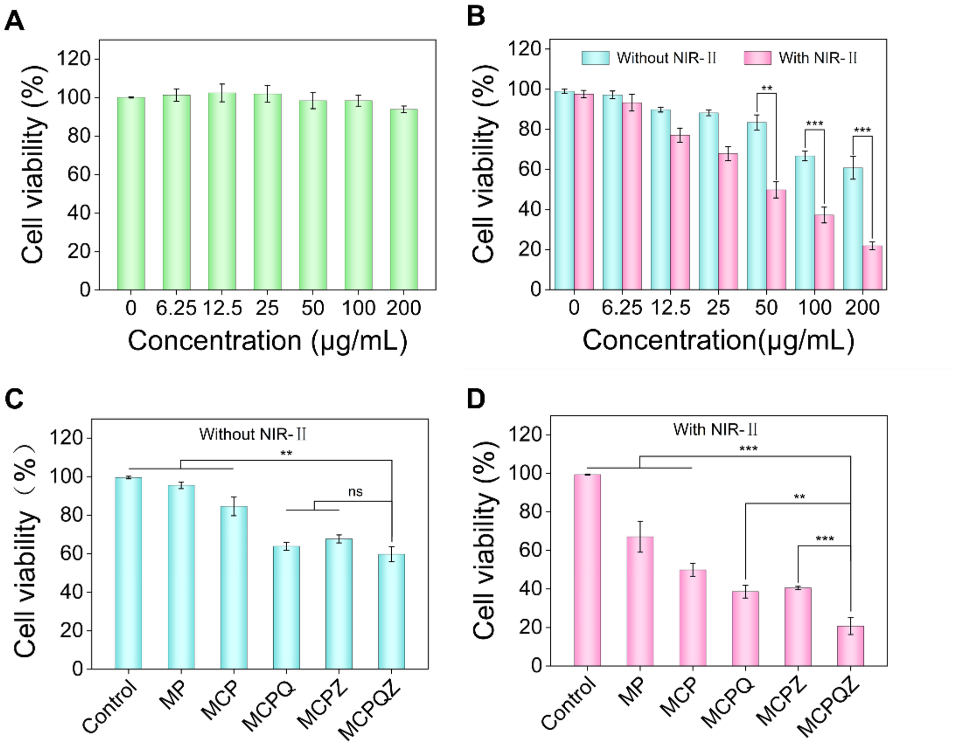


**Figure S23.** (A) Cell viability of H9C2 cells cultured with MCPQZ at different concentrations. The data are presented as mean ± S.D. (n=3). (B) Cell viability of 4T1 cells cultured with MCPQZ at different concentrations with/without 1064 nm laser irradiation (1 W $cm^{-2}$, 5 min). The data are presented as mean ± S.D. (n=3). $^{***}P < 0.001$ for without NIR-II *vs* with NIR-II. (B) Cell viability of 4T1 cells with different treatments without 1064 nm laser irradiation. The data are presented as mean ± S.D. (n=3). $^{**}P < 0.01$ *vs* MCPQZ, ns: no significant difference. (C) Cell viability of 4T1 cells with different treatments with 1064 nm laser irradiation (1 W $cm^{-2}$, 5 min). The data are presented as mean ± S.D. (n=3). $^{**}P < 0.01$, $^{***}P < 0.001$ *vs* MCPQZ + NIR-II.

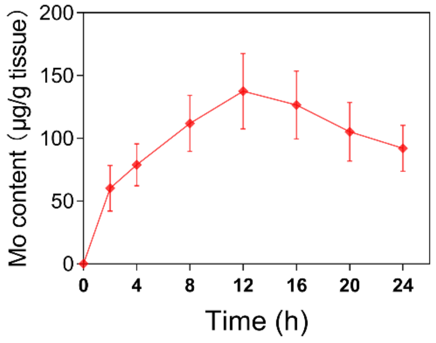


**Figure S24.** The biodistribution of MCPQZ in 4T1 tumor-bearing mice at different time points. The data are presented as mean ± S.D. (n=3).

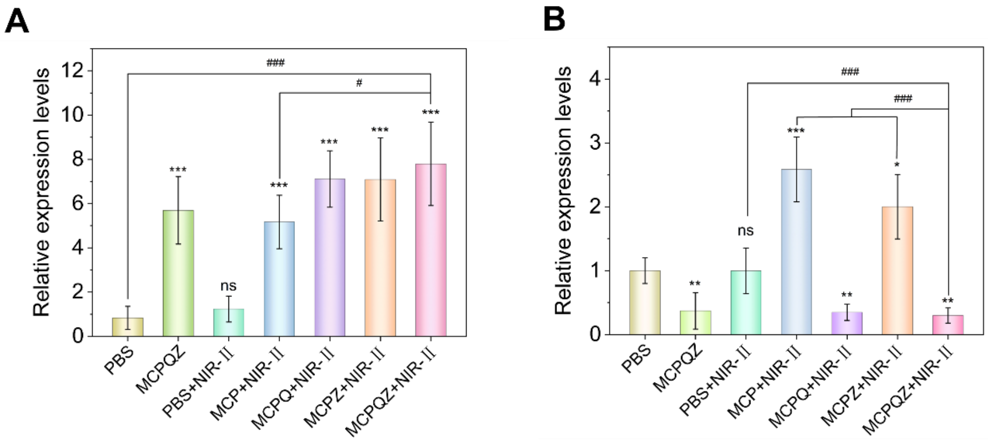


**Figure S25.** Expression of HSP70 and HO-1 in 4T1 cells with different treatments. The data are presented as mean ± S.D. (n=3). $^{*}P$<0.05, $^{**}P$<0.01, $^{***}P$<0.001 *vs* control, $^{\#}$P<0.05, $^{\#\#}P$<0.01, $^{\#\#\#}P$<0.001 *vs* MCPQZ + NIR-II, ns: no significant difference.

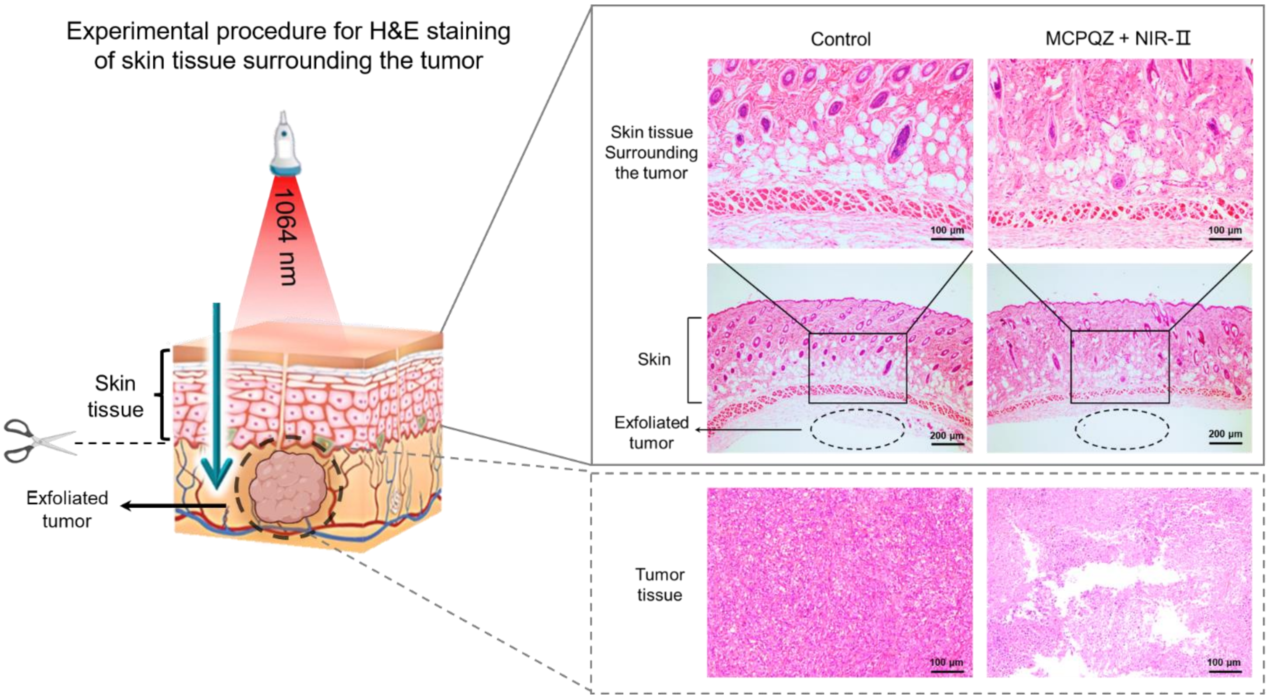


**Figure S26.** Experimental procedure and H&E staining of tumor and its surface skin tissue of 4T1-tumor bearing mice intravenous injected with PBS or MCPQZ (equivalent to 10 mg $MoS_2$ or 8.596 mg QE or 0.269 mg Znpp IX $kg^{-1}$ mice) irradiated with 1064 nm laser (1 W $cm^{-2}$, 5 min).

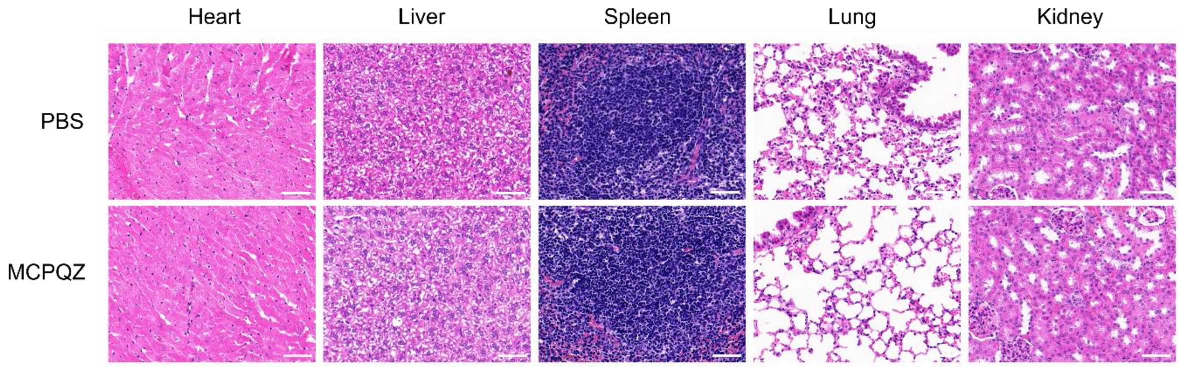

**Figure S27.** H&E staining of major organs (heart, liver, spleen, lung, and kidney) of mice intravenous injected with PBS or MCPQZ (equivalent to 10 mg $MoS_2$ or 8.596 mg QE or 0.269 mg Znpp IX $kg^{-1}$ mice). Scale bar = 20 μm.

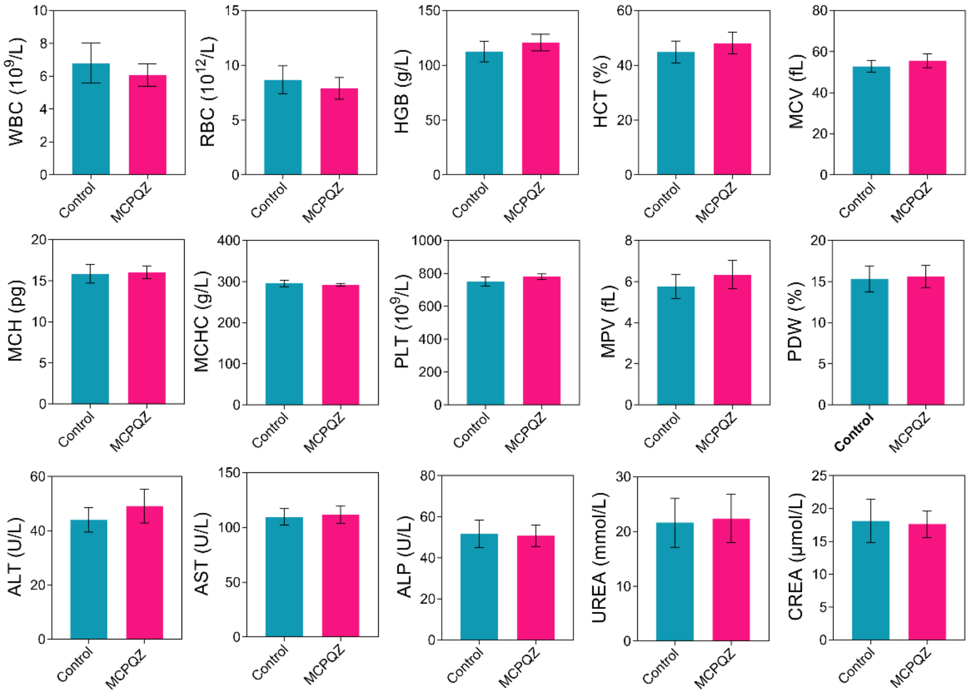


**Figure S28.** Routine blood examination and serum biochemical analysis of mice after intravenous injection of MCPQZ (equivalent to 10 mg $MoS_2$ or 8.596 mg QE or 0.269 mg Znpp IX $kg^{-1}$ mice), including white blood cells (WBC), red blood cells (RBC), hemoglobin (HGB), hematocrit (HCT), mean corpuscular volume (MCV), mean corpuscular hemoglobin (MCH), mean corpuscular hemoglobin concentration (MCHC), red cell distribution width (RDW), platelets (PLT), mean platelet volume (MPV), platelet distribution width (PDW), alanine aminotransferase (ALT), aspartate aminotransferase (AST), alkaline phosphatase (ALP), urea nitrogen (UREA), and creatinine (CREA). The data are presented as mean ± S.D. (n=3).
